# Taxonomic Significance of Seed Morphology in *Veronica* L. (Plantaginaceae) Species from Central Europe

**DOI:** 10.3390/plants11010088

**Published:** 2021-12-28

**Authors:** Małgorzata Mazur, Katarzyna Marcysiak, Agnieszka Dunajska, Magdalena Gawlak, Tomasz Kałuski

**Affiliations:** 1 Faculty of Biological Sciences, Kazimierz Wielki University, Ossolińskich 12, 85-093 Bydgoszcz, Poland; agnieszka1190@wp.pl; 2 Institute of Plant Protection—National Research Institute, Władysława Węgorka 20, 60-318 Poznan, Poland; m.gawlak@iorpib.poznan.pl (M.G.); t.kaluski@iorpib.poznan.pl (T.K.)

**Keywords:** micromorphology, numerical analysis, scanning electron microscopy, seed, seed coat

## Abstract

In this study, 1159 seeds of 29 Central European species of the genus *Veronica* were analyzed based on scanning electron microscopy images. The species belonged to nine subgenera: *Beccabunga*, *Chamaedrys*, *Cochlidiosperma*, *Pellidosperma*, *Pentasepalae*, *Pocilla*, *Pseudolysimachium*, *Stenocarpon* and *Veronica*, following the newest phylogenetic classification of the genus. Nine measured characteristics of seeds and nine ratios were analyzed statistically using ANOVA followed by post hoc testing, cluster analysis and discriminant analysis. In most cases, the results were not congruent with the contemporary classification of the genus. Examinations of qualitative seed features by scanning electron microscopy included the cochlidiospermous or discoid seed type, the seed shape, the general sculpture of the seed coat surface, the sculpture of anticlinal and periclinal walls and some species-specific traits such as the presence of the epidermal appendix. All these features, apart from seed shape, were useful to distinguish all subgenera and some species within subgenera: *Beccabunga*, *Chamaedrys*, *Pellidosperma*, *Pocilla* (only *V. filiformis*) and *Veronica*. The identification key based on the seed micromorphological features was prepared.

## 1. Introduction

The genus *Veronica* L. comprises about 450 species of about 500 belonging to the tribe *Veroniceae* [1,2] of the family Plantaginaceae sensu Angiosperm Phylogeny Group [3], about 39 of which occur in the central part of Europe [4]. Five sections were usually distinguished within the genus [5]: *Veronicastrum* W.D.J. Koch., *Veronica*, *Beccabunga* (Hill) Dumort., *Pocilla* Dumort. and *Pseudolysimachium* W.D.J.Koch. Phylogenetic research, based on DNA analyses of ribosomal ITS-regions and plastid trnL-F region [6,7,8,9] and cpDNA [10], chromosome numbers [11,12], morphological features of plant parts [13,14,15,16], pollen grains morphology [17,18,19,20], chemotaxonomic studies by flavonoid and iridoid glycosides [21,22,23,24] introduced changes in the systematics of the genus *Veronica*. In order to make the genus *Veronica* a monophyletic taxon, the genera *Synthyris* Benth. and *Pseudolysimachion* Opiz were included [1,12]. Albach et al. [1] excluded the genus *Veronicastrum* Heist. ex Fabr. and divided the genus *Veronica* into 13 subgenera: *Veronica*, *Beccabunga* (Hill) M.M. Mart. Ort., Albach & M.A. Fisch., *Pseudolysimachium* (W.D.J. Koch) M.M. Mart. Ort., Albach & M.A. Fisch., *Synthyris* (Benth.) M.M. Mart. Ort., Albach & M.A. Fisch., *Cochlidiosperma* (Rchb.) M.M. Mart. Ort. & Albach, *Pellidosperma* (E.B.J. Lehm.) M.M. Mart. Ort., Albach & M.A. Fisch., *Stenocarpon* (Boriss.) M.M. Mart. Ort., Albach & M.A. Fisch., *Triangulicapsula* M.M. Mart. Ort., Albach & M.A. Fisch., *Pocilla* (Dumort.) M.M. Mart. Ort., Albach & M.A. Fisch., *Pentasepalae* M.M. Mart. Ort., Albach & M.A. Fisch., *Chamaedrys* (W.D.J. Koch) M.M. Mart. Ort., Albach & M.A. Fisch., *Hebe* Comm. ex Juss. and *Derwentia* Raf. Southern hemisphere genera such as *Chionohebe* B.G. Briggs & Ehrend., *Derwentia*, *Detzneria* Diels, *Hebe*, *Hebejeebie* Heads, *Heliohebe* Garn.-Jones, *Leonohebe* Heads and *Parahebe* W.R.B. Oliv. were subsequently recognized as a subgenus *Pseudoveronica* J.B. Armstr. [2]. As a result, there are currently 12 subgenera, including nine of them widespread in temperate climates and sometimes in the mountains on the three continents of the northern hemisphere, one widespread in the western part of North America (*Synthyris*), one containing two single-legged species from Turkey and Spain (*Triangulicapsula*) and the last one containing the previously mentioned species of the southern hemisphere (*Pseudoveronica*).

Because of the parallel evolution of morphology and growth habits of *Veronica* species [16], it is difficult to find uniform morphological characteristics of the subgenera. Muñoz-Centeno et al. [25] demonstrated that some morphological traits, especially the ultrastructure of the seed coat sculpture, supported classifications based on molecular data. The various characteristics of seeds of *Veronica* species have been used in their descriptions and taxonomy for a long time [26,27,28]. Kulpa [29] prepared the first thorough analysis of the seed morphology of 67 *Veronica* species, including their shapes and traits of the testa sculpture. Based purely on light microscopic observations, the author noticed details of the seed surface such as a chalazal plate and a chalazal podium (plateau), as well as an epidermal appendix. This allowed him to delimit eight infrageneric groups based on seed morphology. Further studies were based on SEM images analyses, taking into account the features of the micro-morphological structure of the seed coat sculpture. These works mostly comprised only several species, sampled from a limited area, e.g., from the Mediterranean [19,30], Spain [31], Iran [32], Turkey [33,34] and Poland [35]. For example, Hassan and Khalik [36] investigated seeds of 29 species mainly from Egypt and Europe. Some studies found a relationship between seed morphology and chromosome number in the genus *Veronica* [22,30]. The most comprehensive study was performed by Muňoz-Centeno et al. [25], including 132 species of all subgenera of the genus *Veronica* and using material from the most extensive herbaria in western Europe (France, Germany, the United Kingdom, Spain), Turkey and Japan. Most authors of the above-mentioned studies tended to create groups of taxa based on their testa sculpture or other traits related to the phylogeny of the genus.

The present study covers 29 *Veronica* species common in Central Europe and one of our purposes was to compare seed measurements from different geographical localities. The main purpose of the study was to check whether the relations of species and subgenera, inferred from the qualitative seed features and from the statistical analyses based on quantitative traits, were congruent with the phylogenetic classification by Albach et al. [1] and Albach and Meudt [10], that is, if the analyzed traits had taxonomic importance. To achieve this goal, we took the following steps: (1) We measured the seeds and statistically analyzed the results and (2) assessed the qualitative features of the seeds, with emphasis on the testa surface. Another goal was to assess whether Kulpa’s [29] observations could be confirmed by SEM.

## 2. Results

### 2.1. Seed Feature Assessment

#### 2.1.1. Seed Type

**Figure 1:**
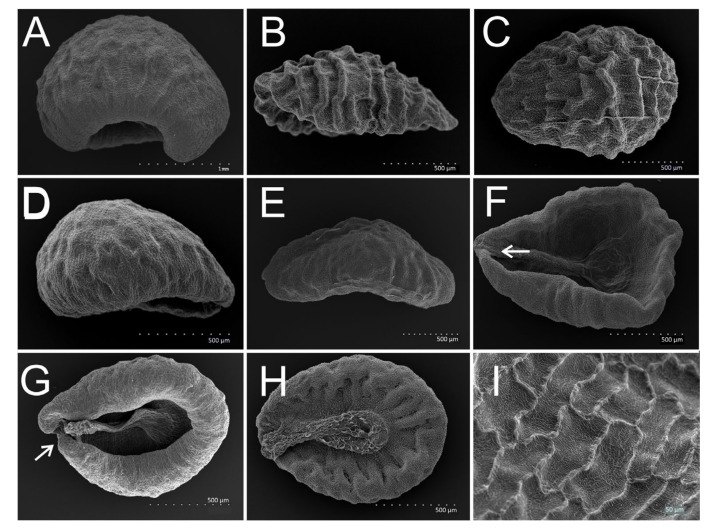
Cochlidiospermous (ventrally concave) seeds of *Veronica* species. (**A**) *V. hederifolia*—deep cochlidiospermous, large seed; (**B**) *V. polita*—shallow cochlidiospermous, small seed, with deeply wrinkled surface; (**C**) *V. persica*—small, obovoid seed with deeply wrinkled surface; (**D**) *V. praecox*—deep cochlidiospermous seed with smooth surface; (**E**) *V. triphyllos*—shallow cochlidiospermous seed with smooth surface; (**F**) *V. triphyllos*—broadly obovoid shape in outline with a trace of micropyle in the form of a shallow pore (arrow); (**G**) *V. praecox*—broadly obovoid seed with a trace of micropyle in the form of a deep pore (arrow); (**H**) *V. filiformis*—broadly obovoid seed with the large chalazal plate raised on the plateau, with large polygonal structures on the chalaza (without papillae), the sculpture of the remaining seed surface is reticulate-verrucate, polygons small; (**I**) *V. sublobata*—reticulate-corrugate sculpture with convex, corrugate anticlinal walls and flat, rugose periclinal walls.

**Figure 2:**
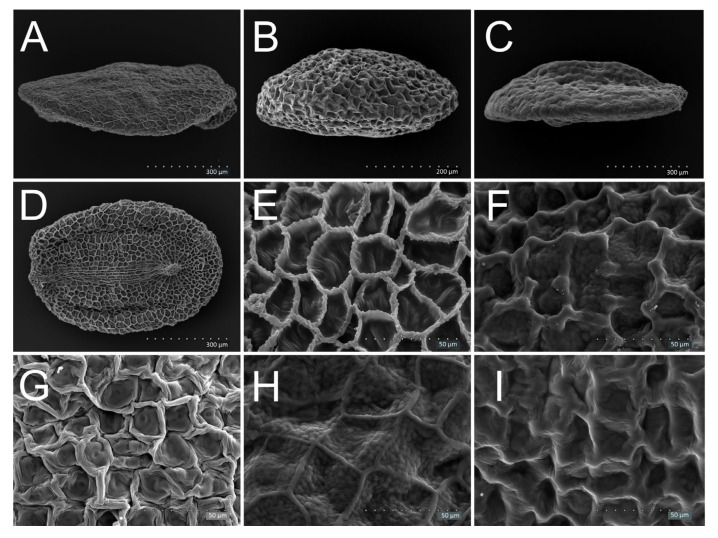
Discoid, small seeds of *Veronica* species; (**A**) *V. longifolia*—discoid-thin seed; (**B**) *V. anagalis-aquatica*—discoid-thick seed; (**C**) *V. peregrina*—discoid-thick seed with a keel; (**D**) *V. longifolia*—elliptical seed with the reticulate-striate sculpture; (**E**) *V. longifolia*—reticulate-striate sculpture with convex, striate anticlinal walls and concave, striate periclinal walls; (**F**) *V. anagalis-aquatica*—reticulate sculpture with convex, rugose anticlinal walls with lumps and slightly colliculate, granulate periclinal walls; (**G**) *V. serpyllifolia*—reticulate-verrucate sculpture with convex and rugose anticlinal walls and rugose periclinal walls, with large papillae; (**H**) *V. beccabunga*—reticulate sculpture with convex anticlinal walls and slightly colliculate, granulate periclinal walls; (**I**) *V. catenata*—reticulate sculpture with convex, rugose anticlinal walls and concave, corrugate periclinal walls.

**Figure 3:**
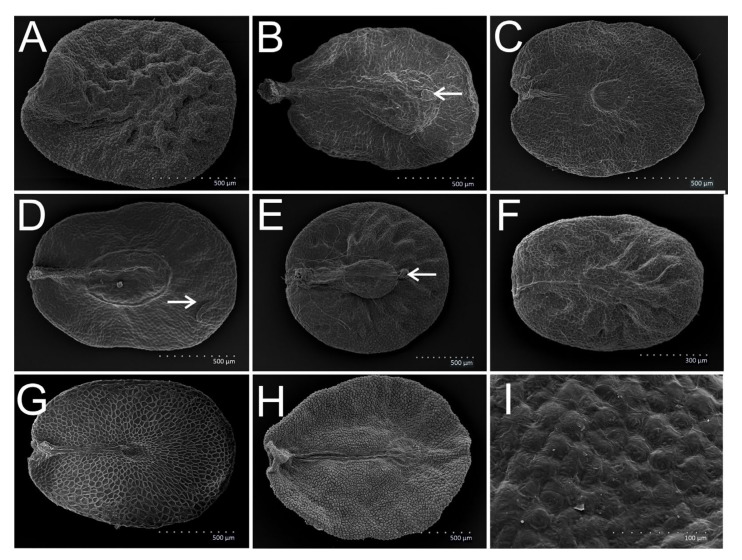
Discoid, large seeds of *Veronica* species; (**A**) *V. austriaca*—broadly elliptical seed with thick but shallow wrinkles on the surface; (**B**) *V. teucrium*—broadly obovoid seed with large chalazal plate and epidermal appendix in the upper part of the chalazal plate (arrow); (**C**) *V. aphylla*—broadly elliptical shape in outline with small chalazal plate; (**D**) *V. chamaedrys*—broadly elliptical seed with large chalazal plate and epidermal appendix in the upper part of the seed, outside chalazal plate (arrow); (**E**) *V. austriaca*—suborbicular shape seed with epidermal appendix in the upper part of the chalazal plate (arrow); (**F**) *V. verna*—broadly elliptical seed with large chalazal plate and no epidermal appendix; (**G**) *V. scutellata*—broadly elliptical shape in outline with chalazal plate at approximately half of the length of the seed; (**H**) *V. fruticans*—broadly elliptical seed with chalazal plate in the upper part of the seed, verrucate seed coat; (**I**) *V. chamaedrys*—reticulate-verrucate-colliculate sculpture with convex, corrugate periclinal walls and flat, rugose anticlinal walls.

**Figure 4:**
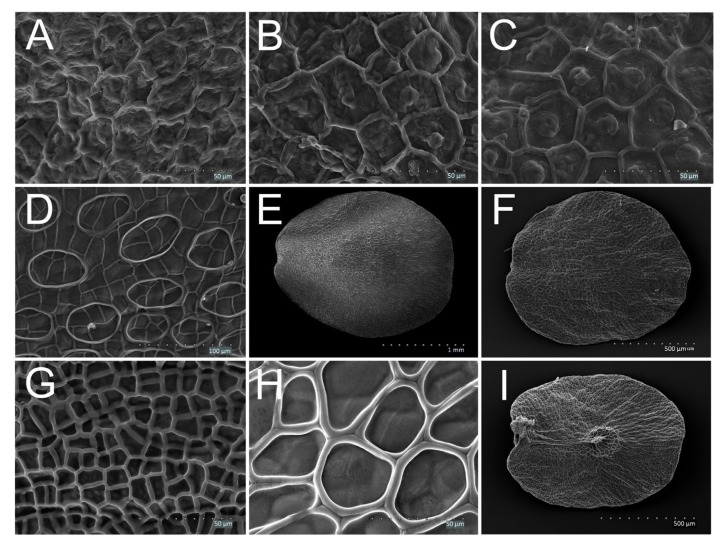
Discoid, large seeds of *Veronica* species; (**A**) *V. arvensis*—reticulate-verrucate sculpture with convex, rugose anticlinal walls and with rugose, corrugate periclinal walls, with papillae not clearly visible; (**B**) *V. dillenii*—reticulate-verrucate sculpture with convex, rugose anticlinal walls and with flat, rugose, corrugate periclinal walls and with papillae clearly visible; (**C**) *V. verna*—reticulate-verrucate sculpture with convex, rugose anticlinal walls and with flat, rugose and granulate periclinal walls, with papillae clearly visible; (**D**) *V. montana*—reticulate sculpture with supracellular rings, with convex anticlinal walls and colliculate periclinal walls; (**E**) *V. montana*—broadly elliptical shape in outline, supracellular rings, seed surface smooth; (**F**) *V. aphylla*—broadly elliptical shape in outline, seed surface wrinkled; (**G**) *V. officinalis*—reticulate sculpture with convex, smooth anticlinal walls and colliculate, granulate periclinal walls, polygonal structures small; (**H**) *V. scutellata*—reticulate sculpture with convex, smooth anticlinal walls and colliculate, smooth periclinal walls, polygons large; (**I**) *V. urticifolia*—broadly elliptical shape in outline with large polygons around the chalaza.

The seed types of all 29 species of 9 subgenera of *Veronica* were assessed (Table 1). Seeds of the subgenera *Pocilla*, *Pellidosperma* and *Cochlidiosperma* were cochlidiospermous (that is ventrally concave) (Figure 1), whereas those of the subgenera *Pseudolysimachium*, *Beccabunga*, *Veronica*, *Chamaedrys*, *Pentasepalae* and *Stenocarpon* were discoid (Figure 2, Figure 3 and Figure 4). In the subgenera *Veronica* and *Stenocarpon*, all seeds were of the discoid-thin type (Table 2; Figure 2A). Most of the seeds of *Chamaedrys*, *Pentasepalae* and *Pseudolysimachium* were also discoid-thin, whereas in *Beccabunga*, all seeds were discoid-thick (Table 2, Figure 2B). Seeds of *V. peregrina* (*Beccabunga*) had a characteristic keel (Figure 2C).

#### 2.1.2. Seed Shape

We recorded all seed shapes, that is, obovoid (Figure 1C), broadly obovoid (Figure 3B), elliptical (Figure 2D), broadly elliptical (Figure 3C) and suborbicular (Figure 3E), in the subgenera *Pseudolysimachium*, *Beccabunga*, *Veronica*, *Chamaedrys*, *Pocilla* and *Pellidosperma* (Table 2). In *Pentasepalae* and *Cochlidiosperma*, obovoid, broadly obovoid, broadly elliptical and suborbicular shapes were observed. In *Stenocarpon*, seeds of only three outline types were present, namely broadly elliptical, broadly obovoid and suborbicular. The broadly elliptical seeds dominated or co-dominated in most species.

#### 2.1.3. Sculpture of Seed Coat Surface

Seven general types of testa surface sculpture were recognized, namely reticulate (Figure 2I), reticulate-striate (Figure 2E), reticulate-verrucate (Figure 2G), reticulate with supracellular rings (Figure 4D), reticulate-corrugate (Figure 1I), reticulate-verrucate-colliculate (only in *V. chamaedrys*, Figure 3I) and verrucate (only in *V. fruticans*, Figure 3H). The reticulate-verrucate sculpture was observed in most of the seeds of the species studied (Table 2). The periclinal walls of the testa surface sculpture were most often concave (Figure 2E,I) or flat (e.g., Figure 4C) and rugose (e.g., Figure 1I and Figure 2G), less frequently smooth (Figure 4H), sometimes also colliculate (Figure 4H) or corrugate (Figure 4A,B) and sometimes also granulate (Figure 2F,H and Figure 4C). Convex walls were rare. Anticlinal walls were rugose (e.g., Figure 2F,G,I) and most often convex (e.g., Figure 2E–I), but sometimes flat or concave.

### 2.2. Statistical Differences among Subgenera and Species

The values of characteristics of individual species and subgenera were mostly unimodally distributed. Characters values distributions in the set of all data deviated from normal, both for raw and standardized data. In the set of means of all individuals, only seed width at ½, ¼ and ¾ to seed length (*W*½/*L*, *W*¼/*L*, *W*¾/*L*), and seed width at ¼ to width at ¾ (*W*¼/*W*¾) were normally distributed. In the set of means of species, most traits were normally distributed, with the exceptions of *W*½, *W*¼ and seed thickness at ½ (*T*½). Morphological features were mutually strongly correlated, and relations between ratios were fewer and weaker.

Subgenera *Beccabunga* and *Pseudolysimachium* had the smallest seeds, whereas the seeds of *Cochlidiosperma* were the largest. The remaining subgenera had medium-sized seeds, and the variation of feature values of species within each subgenus was noticeable (Table 3, Figure S1). Seed length (*L*) and width (*W*½, *W*¼, *W*¾) highly correlated with the perimeter (*P*), and therefore differentiated the subgenera in the same way, as well as, in most cases, the length of the chalaza (*LCh*) and the length and width of the chalazal plate (*LPl*, *WPl*). Seed thickness (*T*½) was important for differentiation of subgenera into groups. The largest group, with very thin seeds, comprised *Beccabunga*, *Chamaedrys*, *Pseudolysimachium*, *Stenocarpon*, *Veronica* and *Pentasepalae*. *Cochlidiosperma* seeds were very thick, whereas seeds of *Pocilla* and *Pellidosperma* were intermediate (Table 3, Figure S1). The values of ratios complemented the characteristics of the subgenera, e.g., *Beccabunga*, with the smallest *LCh* and *LPl* values, had the greatest value of the *LCh/LPl* ratio (Table 3, Figure S1). The values of some traits differed among species within the same subgenus, e.g., *L/T½* in *Beccabunga* and *Veronica* and *LCh/LPl* in *Veronica* (Figure S1).

All examined features differentiated nine subgenera with statistical significance (*p* ≤ 0.01) according to ANOVA and Kruskal-Wallis tests, but the post-hoc tests pointed out that some subgenera differed from others only in terms of a few features (Table S1). The differences among species revealed by Dunn’s test did not fully confirm the above results for subgenera. The differentiation among species of the individual subgenera was more complex. In general, these data were difficult to interpret because of the complex schemes of numerous statistically significant differences (Table S2).

**Figure 5:**
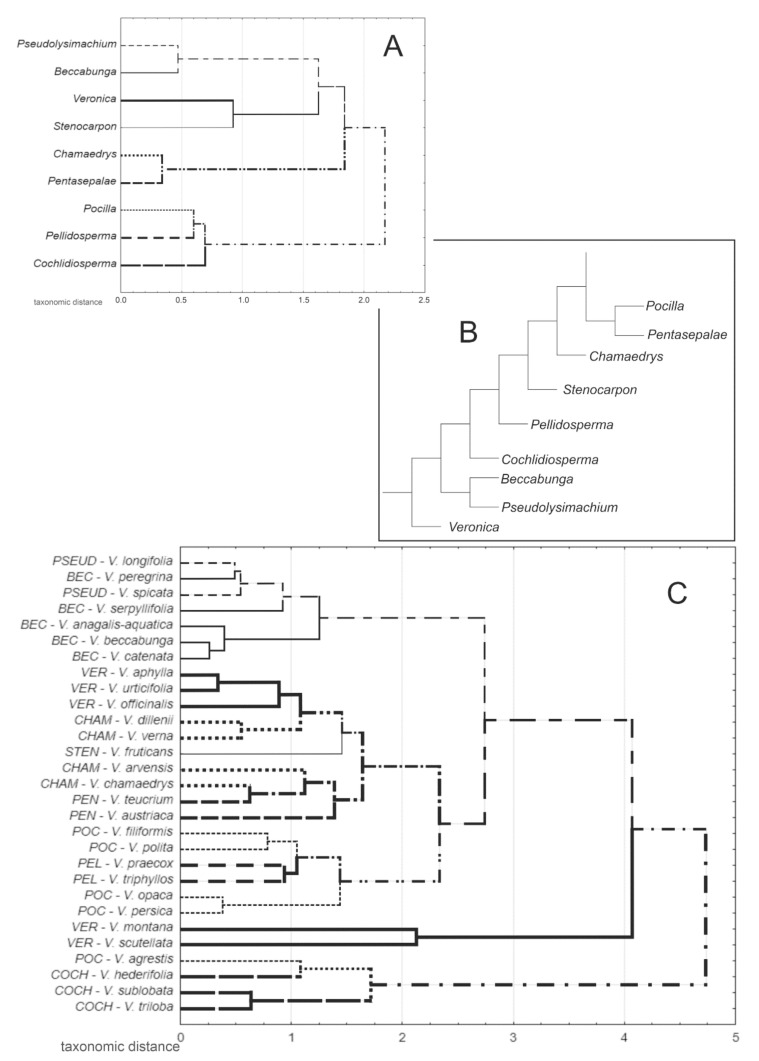
(**A**) UPGMA dendrogram constructed on the shortest Euclidean distances for nine subgenera of the genus *Veronica*, based on quantitative traits; (**B**) simplified tree showing the relationships between the analyzed subgenera of *Veronica*, based on the fragment of combined cpDNA and ITS phylogeny by Albach and Meudt [10]; only the binding order is shown; (**C**) UPGMA dendrogram constructed on the shortest Euclidean distances for 29 species of the genus *Veronica*, based on quantitative traits. Subgenera abbreviations as in Table 1.

Cluster analysis among subgenera separated the cluster comprising *Pocilla*, *Pellidosperma* and *Cochlidiosperma* from others. *Chamaedrys* was close to *Pentasepalae* and *Pseudolysimachium* was close to *Beccabunga* (Figure 5A). The species dendrogram showed differences within the subgenera. The species of *Beccabunga* and *Pseudolysimachium* formed the most compact cluster. The *Cochlidiosperma* species were grouped together and separated from others, but one species of *Pocilla* (*V. agrestis*) clustered with them. The other species of *Pocilla* were mixed in a cluster with *Pellidosperma*. Two species of the subgenus *Veronica*, namely *V. montana* and *V. scutellata*, were highly separated from all others, including other species of this subgenus (Figure 5C). The results of the discrimination analysis support the clustering results. The scatter plot shows the separation of the subgenus *Cochlidiosperma* and similarity of *Beccabunga* to *Pseudolysimachium* and *Pocilla* to *Pellidosperma*. The subgenera *Veronica*, *Chamaedrys* and *Pentasepalae* were also similar (Figure S2A). Species of particular subgenera showed a similar distribution, as in the case of the dendrogram discussed above (Figure S2B). The features, which most significantly discriminated subgenera, were *P*, *LCh/LPl* and *L/T½*, related to the first discrimination variable, and *L/T½*, *LCh/LPl* and *W¼/W¾*, related to the second discrimination variable.

## 3. Discussion

### 3.1. Subgenera and Species—Identification and Relations

#### 3.1.1. Subgenus *Cochlidiosperma* (COCH)—*V. hederifolia*, *V. sublobata*, *V. triloba*

Seeds of this subgenus were highly specific and easy to recognize: They were the biggest and deeply cochlidiospermous with strongly folded edges, which was in agreement with the findings of Kulpa [29]. The general seed coat structure was reticulate-corrugate, as previously demonstrated by Muñoz-Centeno et al. [25]. In our analyses, species of this subgenus formed a separate group but were closest to *Pocilla*. Kulpa [29] also placed them into a separate group. In the previous classification [5], the species of this subgenus belonged to the section *Pocilla*, which indicated the similarity of the present subgenera *Cochlidiosperma* and *Pocilla*. The cpDNA and ITS analyses showed the subgenus *Cochlidiosperma* to be the least supported in *Veronica* and not related to *Pocilla* (Figure 5B) [10]. It was impossible to distinguish the three species examined in our study based on the seed features, and their morphology is generally very similar. In some classifications, *V. sublobata* and *V. triloba* are treated as subspecies of *V. hederifolia* (e.g., [5]).

#### 3.1.2. Subgenus *Pellidosperma* (*PEL*)—*V. praecox*, *V. triphyllos*

These seeds were also cochlidiospermous, but shallower with smooth or only very slightly wrinkled surface, as previously indicated by Kulpa [29]. This author observed the seed edge in subgenus *Pellidosperma* was not or slightly folded. In our study this was true only for *V. triphyllos*, whereas *V. praecox* seed edges were strongly folded, which at the same time helped to distinguish between species. The trace of the micropyle and the incision at the base of a seed were other features observed by Kulpa [29] to discuss. The author defined the trace of the micropyle in the seeds of this subgenus (separate group in his work) as the dark spot at the base on the down edge of the seed, and this observation was not confirmed in the present study. On the other hand, he pointed out the incision at the seed base, which was shallow (*V. triphyllos*) or deep (*V. praecox*), as the important feature to distinguish between species, and this was confirmed in the present study. In our opinion, the incision could be the trace of the micropyle. The reticulate-verrucate seed coat structure we found was consistent with Muñoz-Centeno et al. [25]. Based on the results of the statistical analyses, this subgenus was closest to *Pocilla*, but this finding had not been supported by any previous molecular nor genetic studies [10,22].

#### 3.1.3. Subgenus *Pocilla* (POC)—*V. agrestis*, *V. filiformis*, *V. opaca*, *V. polita*, *V. persica*

Seeds of this subgenus could be easily recognized, however, the identification of the species based on the seed features was difficult. The seeds were medium-sized, cochlidiospermous, with a deeply transversely wrinkled dorsal seed surface and a generally radially wrinkled ventral side. These features were observed, e.g., by Kulpa [29] and Ghimire et al. [37], and confirmed in the present study. The seed coat was reticulate-verrucate [25]. Only *V. filiformis* was easy to recognize among the species of the subgenus, namely by the chalaza raised on the podium (plateau), and this feature has earlier been described by Kulpa [29]. The other four species could not be distinguished, and the features found by Kulpa [29], such as the type of wrinkles, more or less regular or dentate, were not suitable.

In our analyses, seeds of species of this subgenus were most similar to those of *Pellidosperma*, and *V. agrestis* was close to the species of the subgenus *Cochlidiosperma*. In general, our results showed that species with cochlidiospermous seeds, that is, species of the subgenera *Pocilla*, *Cochlidiosperma* and *Pellidosperma*, were close. These results were not consistent with previous genetic and biochemical findings [6,7,9,10,16,22,23]. While some earlier findings indicate the close similarity of *Cochlidiosperma* to *Pellidosperma*, this could have been influenced by the increase in the substitution index used because of their annual growth habit [16]. Phylogenetic studies have shown that the subgenus *Pocilla* is attached to *Pentasepalae* and *Chamaedrys* [9,10,16,23].

#### 3.1.4. Subgenus *Pentasepalae* (PEN)—*V. austriaca*, *V. teucrium*

The two species of the subgenus had large seeds with large chalaza, a well-developed chalazal podium (plateau) and the epidermal appendix situated in the upper part of the chalazal plate. These features were found by Kulpa [29] and confirmed in the present work. Kulpa [29] only analyzed *V. teucrium* but included *V. chamaedrys* (from the subgenus *Chamaedrys*) into the group comprising *V. teucrium*, and the presence of the epidermal appendix was the important common feature. We confirmed that the seed coat was reticulate-verrucate, as observed in the previous works [25]. According to the cluster analysis, the *Pentasepalae* species were close to the *Chamaedrys* and the taxonomic distance between them was the shortest. Phylogenies based on cpDNA and ITS by Albach and Meudt [10] placed this subgenus as a sister clade to *Pocilla*, but not far from *Chamaedrys*. Features distinguishing the species were not found.

#### 3.1.5. Subgenus *Chamaedrys* (CHAM)—*V. arvensis*, *V. chamaedrys*, *V. dillenii*, *V. verna*

The seeds of this subgenus were small, flat, with a large chalazal plate. The presence of the easily visible chalazal plateau was the common feature of the subgenera *Chamaedrys* and *Pentasepalae*, consistent with the study by Kulpa [29]. The seed coat structure was reticulate-verrucate, but in *V. chamaedrys* also colliculate, which was consistent with the findings by Muñoz-Centeno et al. [25]. *Veronica chamaedrys* differed from other species of the subgenus by its smooth seed surface, in contrast to the wrinkled surface of the seeds of other species. The presence of the epidermal appendix situated in the upper part of the seed, outside the chalazal plate, was another important feature of this species. This was in agreement with the findings of Kulpa [29]. In the previous classification, *V. chamaedrys* belonged to the section *Veronicastrum*, whereas the remaining species of the subgenus were included in section *Pocilla* [5].

In our cluster analyses, the subgenus *Chamaedrys* was closest to *Pentasepalae*, as stated above, but species of *Chamaedrys* were mixed with species of *Pentasepalae*, *Veronica* and *Stenocarpon*. These relationships were not in line with any previous findings [1,10,19,30].

#### 3.1.6. Subgenus *Beccabunga* (BEC)—*V. anagalis-aquatica*, *V. beccabunga*, *V. catenata*, *V. peregrina*, *V. serpyllifolia*

The small size of the discoid-type seeds, combined with the reticulate or reticulate-verrucate testa surface sculpture, may be the basis for distinguishing them from other seeds. These seeds were most similar to *Pseudolysimachium*. In former classifications, *V. peregrina* and *V. serpyllifolia* were included into the sections *Pocilla* and *Veronicastrum* (e.g., [5]), respectively. Kulpa [29] integrated these two species into the group comprising some species of the present subgenera *Veronica* and *Stenocarpon*. This author grouped the other species of the present subgenus *Beccabunga* (that is *V. anagallis-aquatica*, *V. peregrina*, *V. catenata*) together with species of *Pseudolysimachium*. In our analyses, *V. peregrina* and *V. serpyllifolia* differed from the remaining species of the subgenus by the reticulate-verrucate type of the testa surface, but this type was characteristic of many subgenera and former sections, including *Pocilla* and *Veronicastrum* [30]. The specific feature of *V. peregrina* was the keel on the dorsal side of the seed, observed also by other authors [25,29,31,37]. The strongly flattened seeds were one of the important characteristics of the group delimited by Kulpa [29], and comprising *V. peregrina* and *V. serpyllifolia*. In our study, the relative thickness of the seeds (in relation to the seed length) was different for these species, compared to the remaining species of this subgenus but similar to that of the species of *Chamaedrys*, *Pentasepalae*, *Veronica* and *Stenocarpon*. Kulpa [29] did not find any features differentiating between *V. anagallis-aquatica* and *V. catenata*, but in our study, the former species differed from all others by lumps present on the anticlinal walls of the testa sculpture. Cluster analysis showed a similarity between *Beccabunga* and *Pseudolysimachium*, which was consistent with the results of previous genetic analyses [10].

#### 3.1.7. Subgenus *Pseudolysimachium* (PSEUD)—*V. longifolia*, *V. spicata*

The seeds of these two species were similar and easy to distinguish from others. They were the smallest and very flat, similar to those of the species of *Beccabunga*., as stated above. Most of the previous researchers obtained similar results, e.g., [29,30,38,39]. The closeness of species of these two subgenera has also been confirmed by applying other methods by Albach and Meudt [10], Müller and Albach [9] and Wang et al. [16]. The seeds of the subgenus *Pseudolysimachium* could be identified on the basis of their reticulate-striate testa surface. According to Hassan and Khalik [36], the strong periclinal walls represented the most significant characteristic of this subgenus, but in our opinion, their striate character was more important. Kulpa [29] pointed out that the chalazal plate was slightly greater for *Pseudolysimachium* species, but according to our research, both the length of the chalazal plate and the ratio of the length of the chalaza to the chalazal plate (*LCh*/*LPl*) indicated its small size (Table 3). The distinguishing between species was impossible. The features pointed by Kulpa [29] were either too general or these observations were not confirmed by SEM images.

#### 3.1.8. Subgenus *Veronica* (VER)—*V. aphylla*, *V. montana*, *V. officinalis*, *V. scutellata*, *V. urticifolia*

Medium-sized, flat seeds with a small chalaza situated in the center of the seed were characteristic for the species of this subgenus, as observed by Kulpa [29] and confirmed in the present study. Features of the testa sculpture helped in distinguishing species. The traits indicated by Kulpa (i.e., the size of the seeds or the relative size of the chalazal plate), were difficult to apply. The supracellular rings of the seed coat sculpture were the most prominent features of species of the subgenus, which is in agreement with the results of Muňoz-Centeno et al. [25]. Based on our statistical analyses, the species *V. montana* and *V. scutellata* were separated from the remaining species of this subgenus, but this fact was not supported by genetic analyses [10].

#### 3.1.9. Subgenus *Stenocarpon* (STEN)—*V. fruticans*

*Veronica fruticans* was previously included in the section *Veronicastrum*, together with e.g., *V. serpyllifolia* (now in *Beccabunga*) and the present subgenus *Veronica* [5]. Based on our analysis, the species was indeed most similar to the subgenus *Veronica*, but in general, species of *Veronica*, *Chamaedrys, Pentasepalae* formed one group with *V. fruticans*. Thus, our results partially support the findings of the genetic analyses by Albach et al. [7] and Müller and Albach [9], who indicated the closeness of *Stenocarpon* to *Chamaedrys*, and Taskova et al. [22], who found it close to *Pocilla* and *Pentasepalae*.

### 3.2. Compliance of Statistical Results with the Veronica Taxonomic Classification

In our study, the species analyzed were a priori grouped into the subgenera according to the new phylogenetic classification by Albach et al. [7] and Albach and Meudt [10]. Numerical analyses enabled the determination of statistically significant differences among them, but in terms of their many characteristics, the species formed groups of several subgenera. The most visible differences were related to seed thickness, which corresponded well with the division of the species into those with cochlidiospermous or discoid seeds, as discussed above. The size of the seeds, i.e., their length and width, was another feature that differentiated species and subgenera into those with large seeds (*Cochlidiosperma*), with very small seeds (*Pseudolysimachium* and *Beccabunga*) and with seeds of intermediate size (all others), but these are also features that are evident without measurements or calculations. Despite statistically significant differences among the subgenera and species, it was not possible to establish a set of taxon characteristics that would allow for their differentiation and identification. The subgenus *Cochlidiosperma* was the only exception.

The groupings of subgenera obtained as a result of multivariate analyzes were not consistent with the recent phylogenetic classification of the genus [10]. We only obtained compliance on the similarity between *Beccabunga* and *Pseudolysimachium* (Figure 5A,B).

### 3.3. Comparison of Seed Sizes from Different Sources

Comparison of the different sizes of *Veronica* seeds from different sources, although generally confirming the similar variations in their length and width, showed also some differences.

Most studies have only published data on seed length and width, and only Kulpa [29] measured seed thickness. The results were published either as the descriptive statistics (mean, minimum, maximum, coefficient of variation and confidence interval at *p* = 0.05) based on examining 30–60 seeds of each taxon [29] or as the mean values with the range minimum–maximum [30,36], based on measuring several seeds. Based on these and previous findings, seed size was generally similar, albeit with some exceptions. The average values of length and width of seeds published by Kulpa [29] were in several cases greater compared to our data (e.g., for *V. hederifolia*, *V. austriaca*, *V. triphyllos*, *V. filiformis* or *V. spicata*), but there were no substantial differences among the seed thickness values (Table S2). Some seeds investigated by Hassan and Khalik [36] were smaller, such as those from *V. hederifolia* or *V. urticifolia*. According to Martínez-Ortega and Rico [30], two species of *Pentasepalae* had different seed sizes compared to previously published data, and *V. austriaca* seeds were considerably smaller, whereas seeds of *V. teucrium* were substantially larger (Table S3).

The differences found were most likely due to different sampling locations or collection years. Kulpa [29] obtained a significant part of the research material from herbaria in eastern Germany (former GDR). Martínez-Ortega and Rico [30] mostly analyzed seeds from Spain, whereas seeds of *V. austriaca* and *V. teucrium* were obtained from the old 19th-century collections from Germany and Switzerland, respectively.

## 4. Materials and Methods

### 4.1. Materials

We analyzed material collected from 166 herbarium specimens from herbaria KRA, TRN and BYDG in Poland (Table S1) from 29 species of nine subgenera of the genus *Veronica*, following the newest phylogenetic classification by Albach et al. [1] and Albach & Meudt [10]. Only undamaged, fully developed seeds from matured capsules and from well-preserved herbarium specimens were selected for the examinations. In total, 1159 seeds were investigated, including for comparison seeds of the former section *Pocilla*, used in a previous study (Table 1) [35]. Dry seeds were attached to table mounts using double sided adhesive carbon discs and then were sputter coated with gold. Seed observations were performed using an S-3000N Hitachi Scanning Electron Microscope (SEM). The images were obtained in the Institute of Plant Protection in Poznań, according to the procedure described in Mazur et al. [35]. Seeds of each species were photographed from ventral, dorsal and lateral sides, with magnification from ×35 to ×300 depending on the seed size. We examined 462 images of the dorsal side, 453 of the ventral side and 244 of the lateral side. In this number, we included 125 images of the dorsal side, 184 of the ventral side and 113 of the lateral side from a previous study [35], the measurements of which were made anew. Subsequently, images of the testa sculpture of the 1159 seeds were observed at magnification ×1000, including 422 images prepared in a previous study [35]. In total, 2318 images were examined.

### 4.2. Methods

#### 4.2.1. Qualitative Seed Feature Assessment

The examination was focused on the assessment of features of the testa surface; in addition, several other traits indicated as significant by Kulpa [29] were investigated. For each seed, we determined whether it was cochlidiospermous (that is, ventrally concave) or discoid [25,28,29,31,32,38] but with adaptations [35] to increase objectivity. The discoid (*D*) type of seeds: A thick discoid (*D-tk*) when the value of *L*/*T*½ was less than 3.0 and a thin discoid (*D-tn*) with *L*/*T*½ exceeding or equaling 3.0. The cochlidiospermous (*C*) type of seeds: Deep-cochlidiospermous (*C-d*) when the value of *L*/*T*½ was less than 2.0 and shallow-cochlidiospermous (*C-s*) with this value exceeding or equaling 2.0.

We also used the improved procedure of the evaluation of the seed shape in outline to increase objectivity [35]. We followed the nomenclature used previously [29,31,38,40], where the orbicular, obovoid and elliptical outlines of seeds were named. Additionally, we defined the shapes as follows: Suborbicular (*S*), when the values of *W*¼/*L*, *W*½/*L* and *W*¾/*L* exceeded 0.80, obovoid (*O*), when the value of *W*¼/*W*¾ ranged from 0.9 to of 1.1 and elliptical (*E*), when this value was less than 0.9 or more than 1.1. Additionally, we adopted two intermediate shapes: Broadly obovoid (*BO*) and broadly elliptical (*BE*), when obovoid or elliptical seeds had *W*½/*L* and *W*¼/*L* values or *W*½/*L* and *W*¾/*L* values exceeding or equaling 0.67.

Different seed coat surface types were recognized, and the sculpture of anticlinal and periclinal walls of the testa surface was described, using the terminology applied previously (e.g., [19,25,26,30,41]). The applicability and usefulness of the features used by Kulpa [29] were verified. The key to recognizing the examined subgenera of *Veronica*, and species within the subgenera, was prepared, based on the features indicated by Kulpa [29] and, where possible, on the testa surface features.

#### 4.2.2. Seed Measurements

We measured nine seed characteristics, namely seed perimeter (*P*), maximum length (*L*), width measured at 25% (*W*¼), at 50% (*W*½) and at 75% (*W*¾) of the length, thickness measured at 50% of the length (*T*½) and three characteristics of the chalazal part of seeds, namely the length of the chalazal part (*LCh*) and the length (*LPl*) and the width (*WPl*) of the chalazal plate. Seeds on the images were measured automatically with the use of the digiShape software [42]. Another nine features were obtained as a result of calculations (Table 3). The characteristics analyzed have been developed for the testing of *Veronica* seeds in our earlier work [35].

#### 4.2.3. Statistical Analyses of Quantitative Traits

The normality of the distribution of feature values was tested using Shapiro-Wilks’ test, performed for each species, subgenus and all data, as well as for sets of individual means and species means [43]. The descriptive statistics of 18 morphological seed characteristics were prepared for species and subgenera. Spearman’s rank correlation coefficient was used to check relations among feature values, as many traits were non-parametric [44]. Prior to analysis, data were standardized using the standardization procedure available in STATISTICA [45]. The homogeneity of variance was determined using Levene’s test. Differences between subgenera and species were determined with ANOVA, followed by Tukey’s test; Kruskal-Wallis one-way analysis of variance by ranks [46] with the post-hoc Dunn’s test [47] was used in the case of non-parametric traits. The results are illustrated via box-and-whiskers plots. Multivariate comparisons were performed via cluster analysis [43,48] and discriminant function analysis [49]. These analyses were based on the set of species means. Not strongly mutually correlated features with normal distribution were used, that is *P*, *W*½/*L*, *W*¼/*W*¾, *L*/*T*½, *LCh*/*L*, *LCh*/*LPl* and *LPl*/*WPl*. For all analyses, the software package STATISTICA 13 [45] was applied.

## 5. Conclusions

Referring to the selected examined species we can conclude that numerical taxonomy of analyzed traits of *Veronica* seeds was not helpful in distinguishing subgenera and species. Seed shape did not differentiate subgenera and species. However, the set of qualitative features comprising the seed type and structures of the seed coat surface was useful, especially in distinguishing subgenera and, in many cases, species within subgenera, delimited according to Albach et al. [7] and Albach and Meudt [10], making this set taxonomically important. The features observed by Kulpa [29] were mostly confirmed via SEM images, although their taxonomic importance was sometimes limited.

### The Key to Recognizing the Examined Veronica Species Based on Seed Micromorphological Features

1. Key to subgenera

1ACochlidiospermous seeds (Figure 1)21BDiscoid seeds (Figure 2)42AVery deeply cochlidiospermous, large seeds—more than 1.8 mm long (Figure 1A)
*Cochlidiosperma*
2BLength of seeds up to 1.8 mm (Figure 1B,C)33ASeed surface deeply wrinkled (Figure 1B,C)
*Pocilla*
3BSeed surface smooth (Figure 1D,E), a trace of micropyle in the form of a pore (Figure 1F,G)
*Pellidosperma*
4ASmall seeds—length of seeds up to 1.0 mm (Figure 2A,D)54BLarger seeds—length of seeds more than 1.0 mm (Figure 3A–H and Figure 4E,F)65AReticulate-striate seed coat sculpture, with striate anticlinal and periclinal walls (Figure 2D,E)
*Pseudolysimachium*
5BReticulate or reticulate-verrucate seed coat sculpture, walls not striate (Figure 2F–I)
*Beccabunga*
6AIrregular, thick but shallow wrinkles on the seed surface (Figure 3A); large chalazal plate, with a length more than 30% of the seed length (Figure 3B)76BSmooth or slightly wrinkled seed surface (Figure 4E,F); small chalazal plate with length up to 25% of the seed length (Figure 3C)86CSmooth or slightly wrinkled seed surface, large chalazal plate with length more than 30% of the seed length, epidermal appendix in the upper part of the seed, outside chalazal plate (Figure 3D)
*V. chamaedrys*
7AEpidermal appendix in the upper part of chalazal plate (Figure 3B,E)
*Pentasepalae*
7BEpidermal appendix absent (Figure 3C,F)
*Chamaedrys*
8AChalazal plate at approximately half of the length of the seed (Figure 3G), supracellular rings often occur, at least in the area of the chalazal plate (Figure 4D,E,I)
*Veronica*
8BChalazal plate in the upper part of the seed (Figure 3H)*Stenocarpon*—*V. fruticans*

2. Keys to species within subgenera

*Cochlidiosperma*


It is not possible to distinguish species of the subgenus Cochlidiosperma based on seed characteristics*V. hederifolia*, *V. sublobata*, *V. triloba*
*Pocilla*

1ALarge chalazal plate, chalaza raised on the podium (plateau), with large polygonal structures and a reticulate sculpture, the sculpture of the remaining part of the seed reticulate-verrucate, polygons smaller (Figure 1H)
*V. filiformis*
1BSeeds with other characteristics; it is not possible to further distinguish species of the genus Pocilla based on seed characteristics*V. agrestis*, *V. opaca*, *V. persica*, *V. polita*
*Pellidosperma*

1ASlightly irregularly wrinkled seed surface, with a longitudinal rib on the dorsal side, seed edge not folded; a trace of micropyle (a pore) shallow (Figure 1F)
*V. triphyllos*
1BSlightly irregularly wrinkled seeds with the edge strongly folded; a trace of micropyle (a pore) deep (Figure 1G)
*V. praecox*

*Pseudolysimachium*


It is not possible to distinguish species of the subgenus Pseudolysimachium based on seed characteristics*V. longifolia*, *V. spicata*
*Beccabunga*

1AA distinct keel on the dorsal side of the seed (Figure 2C)
*V. peregrina*
1BSeeds without a keel (Figure 2B)22AAnticlinal walls with lumps (Figure 2F)
*V. anagallis-aquatica*
2BSeeds with other characteristics33AReticulate-verrucate seed coat sculpture; large papillae occupy the entire surface of the periclinal wall, connected with anticlinal walls, their surface and surface of anticlinal walls strongly rugose (Figure 2G)
*V. serpyllifolia*
3BReticulate seed coat structure (Figure 2H,I)*V. beccabunga*, *V. catenata*
*Pentasepalae*


It is not possible to distinguish species of the subgenus Pentasepalae based on seed characteristics*V. austriaca*, *V. teucrium*
*Chamaedrys*

1AReticulate-verrucate seed coat sculpture, papillae usually not clearly visible (Figure 4A)
*V. arvensis*
1BReticulate-verrucate seed coat sculpture, papillae small, usually clearly visible (Figure 4B,C)*V. dillenii*, *V. verna*
*Veronica*

1ANo supracellular rings or rings occurring only singly at the area of chalazal plate, polygonal structures of the testa surface small, their anticlinal walls simple (Figure 4G)
*V. officinalis*
1BReticulate seed coat sculpture with supracellular rings or polygons, at least at the area of the chalazal plate (Figure 4D,E,H,I)22ASupracellular rings or polygons always only on the ventral side, at least near chalaza (Figure 4I)
*V. urticifolia*
2BRings or polygons cover the whole seed surface (Figure 4E)33ARings in the form of regular network of polygons over the colliculate structure of seeds; polygonal structures large, each anticlinal wall consists of the walls of adjacent polygons; the boundary between them is visible (Figure 4H)
*V. scutellata*
3BRings with other characteristics, usually round, more or less regular44ADorsal surface of seeds smooth, without wrinkles (Figure 4E)
*V. montana*
4BDorsal surface of seeds with wrinkles (Figure 4F)
*V. aphylla*



## Figures and Tables

**Table 1:** The number of individuals and seeds analysed of nine subgenera and twenty-nine species of genus *Veronica*.

Subgenus	Subgenus Abbreviation	Species	Species Abbreviation	Number of Individuals	Number of Seeds
*Pseudolysimachium*	PSEUD	*V. longifolia*	long	5	40
		*V. spicata*	spic	4	26
*Beccabunga*	BEC	*V. anagalis-aquatica*	an-a	7	47
		*V. beccabunga*	becc	7	43
		*V. catenata*	cat	2	11
		*V. peregrina*	per	4	27
		*V. serpyllifolia*	serp	3	22
*Veronica*	VER	*V. aphylla*	aph	3	21
		*V. montana*	mont	4	21
		*V. officinalis*	off	7	35
		*V. scutellata*	scut	7	33
		*V. urticifolia*	urti	3	17
*Chamaedrys*	CHAM	*V. arvensis*	arv	7	47
		*V. chamaedrys*	cham	7	40
		*V. dillenii*	dill	8	56
		*V. verna*	vern	5	35
*Pentasepalae*	PEN	*V. austriaca*	aust	3	20
		*V. teucrium*	teuc	2	11
*Stenocarpon*	STEN	*V. fruticans*	frut	6	38
*Pocilla*	POC	*V. agrestis*	agr	11	82
		*V. filiformis*	fili	2	13
		*V. opaca*	opac	11	88
		*V. persica*	pers	14	118
		*V. polita*	pol	10	85
*Pellidosperma*	PEL	*V. praecox*	prae	6	51
		*V. triphyllos*	triph	5	31
*Cochlidiosperma*	COCH	*V. hederifolia*	hed	7	47
		*V. sublobata*	subl	5	45
		*V. triloba*	tril	1	9
		Total		166	1159

**Table 2:** Seed type and shape, general seed coat sculpture and type of anticlinal and periclinal walls of the testa surface of twenty-nine species of nine subgenera of genus *Veronica*. Subgenera abbreviations as in Table 1. *D-tn*—discoid-thin. *D-tk*—discoid-thick. *C-s*—shallow cochlidiospermous. *C-d*—deeply cochlidiospermous. *E*—elliptical. *BE*—broadly elliptical. *O*—obovoid. *BO*—broadly obovoid. *S*—suborbicular.

	Species	Seed Type	Seed Shape	General Sculpture	Periclinal Cell Walls		Anticlinal Cell Walls	
					Sculpture	Bulging State	Sculpture	Bulging State
PSEUD	*V. longifolia*	*D-tn*, *D-tk*	*E*, *BE*, *O*, *BO*, *S*	reticulate-striate	striate	concave/flat	striate	convex
	*V. spicata*	*D-tk*	*E*, *BE*, *O*, *BO*					
BEC	*V. anagalis-aquatica*	*D-tk*	*E*, *BE*, *O*, *BO*, *S*	reticulate	rugose/ corrugate/ granulate	flat/concave/ slightly colliculate	rugose/ rugose with lumps	convex
	*V. beccabunga*	*D-tk*	*E*, *BE*, *O*, *BO*, *S*	reticulate			rugose	convex
	*V. catenata*	*D-tk*	*E*, *BE*, *BO*, *S*	reticulate			rugose	convex
	*V. peregrina*	*D-tk*	*E*, *BE*, *O*, *BO*	reticulate-verrucate			rugose	convex/flat/ concave
	*V. serpyllifolia*	*D-tk*	*E*, *BE*, *O*	reticulate-verrucate			rugose	convex
VER	*V. aphylla*	*D-tn*	*BE*, *S*	reticulate with supracellular rings	smooth/rugose	colliculate	rugose/ rugose with lumps	convex
	*V. montana*	*D-tn*	*E*, *BE*, *BO*, *S*	reticulate with supracellular rings	rugose/corrugate/granulate		smooth/rugose/granulate	
	*V. officinalis*	*D-tn*	*E*, *BE*, *BO*, *S*	reticulate	smooth/rugose/granulate		smooth/rugose/ with lumps	
	*V. scutellata*	*D-tn*	*E*, *BE*, *BO*, *S*	reticulate	smooth/rugose/granulate		smooth/rugose	
	*V. urticifolia*	*D-tn*	*BE*, *O*, *BO*, *S*	reticulate with supracellular rings	smooth/rugose/granulate		smooth/rugose/granulate; always with lumps	
CHAM	*V. arvensis*	*D-tn*, *D-tk*	*E*, *BE*, *O*	reticulate-verrucate	rugose/corrugate	flat/concave	rugose	convex
	*V. chamaedrys*	*D-tn*	*E*, *BE*, *O*, *BO*, *S*	reticulate-verrucate-colliculate	rugose/corrugate/granulate	flat/convex		convex/flat/ concave
	*V. dillenii*	*D-tn*	*BE*, *BO*, *S*	reticulate-verrucate	rugose/corrugate/granulate	flat/colliculate		convex
	*V. verna*	*D-tn*	*E*, *BE*, *O*, *BO*	reticulate-verrucate	rugose/granulate	flat/colliculate		convex
PEN	*V. austriaca*	*D-tn*, *D-tk*	*BE*, *BO*, *S*	reticulate-verrucate	rugose	flat/colliculate	rugose	convex/ concave
	*V. teucrium*	*D-tn*	*BE*, *O*, *BO*, *S*					
STEN	*V. fruticans*	*D-tn*	*BE*, *BO*, *S*	verrucate	rugose	convex	rugose	concave/ flat
POC	*V. agrestis*	*C-s*, *C-d*	*E*, *BE*, *O*, *BO*, *S*	reticulate-verrucate	rugose	flat/concave	rugose	convex
	*V. filiformis*	*C-s*, *C-d*	*E*, *BE*, *BO*, *S*					
	*V. opaca*	*C-s*, *C-d*	*E*, *BE*, *O*, *BO*, *S*					
	*V. persica*	*C-s*, *C-d*	*E*, *BE*, *O*, *BO*, *S*					
	*V. polita*	*C-s*, *C-d*	*E*, *BE*, *O*, *BO*, *S*					
PEL	*V. praecox*	*C-s*, *C-d*	*E*, *BE*, *O*, *BO*, *S*	reticulate-verrucate	rugose	flat/concave	rugose	convex
	*V. triphyllos*	*C-s*, *C-d*	*O*, *BO*, *S*					
COCH	*V. hederifolia*	*C-s*, *C-d*	*BE*, *O*, *BO*, *S*	reticulate-corrugate	rugose	flat/concave	corrugate	convex
	*V. sublobata*	*C-s*, *C-d*	*BE*, *S*					
	*V. triloba*	*C-d*	*BE*, *S*					

**Table 3:** Means and standard deviations of eighteen characters of seeds of nine subgenera of the genus *Veronica*. Subgenera abbreviations as in Table 1.

Characters of Seeds	Character Abbreviation	PSEUD	BEC	VER	CHAM	PEN	STEN	POC	PEL	COCH
Perimeter [mm]	*P*	2.16 ± 0.35	1.84 ± 0.40	4.32 ± 1.30	3.57 ± 0.73	4.54 ± 1.12	4.30 ± 0.36	5.27 ± 1.07	3.61 ± 0.88	7.82 ± 1.33
Length [mm]	*L*	0.69 ± 0.12	0.63 ± 0.15	1.35 ± 0.37	1.15 ± 0.18	1.40 ± 0.25	1.32 ± 0.13	1.54 ± 0.27	1.18 ± 0.24	2.28 ± 0.31
Width at ½ [mm]	*W*½	0.48 ± 0.09	0.44 ± 0.09	1.08 ± 0.30	0.86 ± 0.18	1.13 ± 0.23	1.09 ± 0.11	1.09 ± 0.22	0.91 ± 0.22	1.96 ± 0.33
Thickness at ½ [mm]	*T*½	0.22 ± 0.04	0.22 ± 0.03	0.25 ± 0.07	0.28 ± 0.05	0.38 ± 0.08	0.30 ± 0.05	0.72 ± 0.18	0.60 ± 0.08	1.37 ± 0.28
Width at ¼ [mm]	*W*¼	0.39 ± 0.09	0.38 ± 0.09	0.95 ± 0.24	0.73 ± 0.17	1.06 ± 0.18	0.95 ± 0.10	0.87 ± 0.20	0.76 ± 0.24	1.71 ± 0.31
Width at ¾ [mm]	*W*¾	0.41 ± 0.10	0.37 ± 0.09	0.90 ± 0.26	0.72 ± 0.16	1.00 ± 0.19	0.92 ± 0.14	1.00 ± 0.20	0.80 ± 0.22	1.71 ± 0.32
Length of chalaza [mm]	*LCh*	0.53 ± 0.09	0.45 ± 0.08	0.81 ± 0.18	0.73 ± 0.13	1.00 ± 0.15	0.88 ± 0.10	1.00 ± 0.22	0.74 ± 0.19	1.36 ± 0.23
Length of chalazal plate [mm]	*LPl*	0.15 ± 0.03	0.14 ± 0.03	0.28 ± 0.09	0.41 ± 0.10	0.55 ± 0.13	0.24 ± 0.04	0.49 ± 0.09	0.30 ± 0.07	0.68 ± 0.14
Width of chalazal plate [mm]	*WPl*	0.14 ± 0.03	0.12 ± 0.03	0.22 ± 0.07	0.26 ± 0.09	0.39 ± 0.08	0.21 ± 0.04	0.33 ± 0.06	0.28 ± 0.08	0.51 ± 0.11
	*P*/*L*	3.13 ± 0.38	3.01 ± 0.41	3.16 ± 0.39	3.14 ± 0.52	3.31 ± 0.62	3.26 ± 0.26	3.45 ± 0.42	3.07 ± 0.35	3.50 ± 0.73
Calculated characters–ratios	*W*½/*L*	0.69 ± 0.11	0.71 ± 0.08	0.79 ± 0.06	0.74 ± 0.08	0.81 ± 0.11	0.82 ± 0.07	0.71 ± 0.09	0.77 ± 0.08	0.85 ± 0.08
	*W*¼/*L*	0.55 ± 0.10	0.62 ± 0.09	0.70 ± 0.07	0.62 ± 0.09	0.72 ± 0.08	0.71 ± 0.05	0.57 ± 0.10	0.63 ± 0.11	0.73 ± 0.07
	*W*¾/*L*	0.58 ± 0.12	0.59 ± 0.09	0.66 ± 0.06	0.61 ± 0.07	0.69 ± 0.13	0.69 ± 0.05	0.65 ± 0.08	0.66 ± 0.10	0.73 ± 0.07
	*W*¼/*W*¾	0.98 ± 0.14	1.05 ± 0.14	1.06 ± 0.10	1.03 ± 0.18	1.08 ± 0.21	1.04 ± 0.12	0.88 ± 0.12	0.96 ± 0.23	1.01 ± 0.09
	*L*/*T*½	3.04 ± 0.33	2.82 ± 0.80	4.84 ± 1.11	3.91 ± 0.62	3.78 ± 1.12	4.16 ± 0.46	2.19 ± 0.52	1.94 ± 0.24	1.65 ± 0.18
	*LCh*/*L*	0.75 ± 0.07	0.73 ± 0.08	0.60 ± 0.05	0.63 ± 0.06	0.69 ± 0.10	0.67 ± 0.06	0.64 ± 0.08	0.62 ± 0.08	0.58 ± 0.06
	*LCh*/*LPl*	3.62 ± 0.64	3.22 ± 0.51	3.02 ± 0.55	1.80 ± 0.31	1.86 ± 0.33	3.63 ± 0.44	2.19 ± 0.32	2.48 ± 0.42	2.06 ± 0.40
	*LPl*/*WPl*	1.13 ± 0.19	1.20 ± 0.20	1.28 ± 0.16	1.63 ± 0.32	1.40 ± 0.15	1.17 ± 0.16	1.50 ± 0.23	1.09 ± 0.23	1.37 ± 0.30

## Data Availability

The data presented in this study are available from corresponding authors upon request.

## Supplementary Materials

The following are available online at https://www.mdpi.com/article/10.3390/plants11010088/s1, Figure S1: Box-and-whiskers plots of traits important for differentiating the 29 species of the genus *Veronica*; Point—median; Box—quartiles 25–75%; Whiskers—minimum-maximum; *P*—seed perimeter; *W*½—seed width measured at half of seed length; *T*½—seed thickness measured at half of seed length; *LPl*—length of chalazal plate; *L*/*T*½—ratio of seed length to seed thickness; *LCh*/*LPl*—ratio of chalaza length to length of chalazal plate. Subgenera and species abbreviations as in Table 1, Figure S2: Results of the discriminant analyses on the scatter-plots between the first two variables U_1_ and U_2_; (**A**) for nine subgenera of the genus *Veronica*; (**B**) for 29 species of the genus *Veronica*. Species abbreviations as in Table 1, Table S1: Characteristics differing among the nine subgenera of *Veronica*; results of the post-hoc Dunn’s test with *p* ≤ 0.01. Subgenera abbreviations as in Table 1. Character abbreviations as in Table 3, Table S2: The number of characters differing pairs of twenty-nine species of genus *Veronica* of nine subgenera; the result of the post-hoc Dunn’s test. Subgenera abbreviations as in Table 1, Table S3: Comparisons of seed size of *Veronica* species analyzed in the present study and by Kulpa [29], Martinez-Ortega & Rico [30], and Hassan & Khalik [36]; bold—greatest values for the given species; italics—smallest values for the given species, Table S4: List of herbarium specimens of the 29 *Veronica* species used in the study.
