# Supplementary material for: Taxonomic Significance of Seed Morphology in Veronica L. (Plantaginaceae) Species from Central Europe
Source: Plants (Basel). 2021 Dec 28;11(1):88. doi: 10.3390/plants11010088 (PMC8747532; doi:10.3390/plants11010088)
Supplement: Supplementary file 1 [file plants-11-00088-s001.zip › Table S1.pdf]

Table S1. Characters differing nine subgenera of *Veronica*, the results of the post-hoc Dunn's test, with *p*≤0.01. Subgenera abbreviations as in Table 3. Character abbreviations as in Table 2.

	PSEUD	***BEC***	***VER***	***CHAM***	***PEN***	***STEN***	***POC***	***PEL***
BEC	-							
VER	*P L W½ W¼ W¾ LCh LPl WPl W½/L W¼/L W¾/L LCh/L*	*P L W½ W¼ W¾ LCh LPl WPl P/L W½/L W¼/L W¾/L LCh/L*						
CHAM	*P L W½ W¼ W¾ LCh LPl WPl LCh/L LCh/LPl LPl/WPl*	*P L W½ W¼ W¾ LCh LPl WPl LCh/L LCh/LPl LPl/WPl*	*P L W½ W¼ W¾ LPl W½/L W¼/L W¾/L LCh/LPl LPl/WPl*					
PEN	*P L W½ W¼ W¾ LCh LPl WPl W½/L W¼/L LCh/LPl LPl/WPl*	*P L W½ W¼ W¾ LCh LPl WPl P/L W½/L W¼/L LCh/LPl*	*LPl WPl LCh/L LCh/LPl*	*P L W½ W¼ W¾ LCh WPl*				
STEN	*P L W½ W¼ W¾ LCh W½/L W¼/L W¾/L*	*P L W½ W¼ W¾ LCh WPl P/L W½/L W¼/L W¾/L*	*LCh/L*	*P W½ W¼ W¾ LPl P/L W½/L W¼/L W¾/L LCh/LPl LPl/WPl*	*LPl WPl LCh/LPl*			
POC	*P L W½ W¼ W¾ LCh LPl WPl P/L W¼/W¾ L/T½ LCh/L LCh/LPl LPl/WPl*	*P L W½ W¼ W¾ LCh LPl WPl P/L W¼/L W¾/L W¼/W¾ LCh/L LCh/LPl LPl/WPl*	*P L W¾ LCh LPl WPl P/L W½/L W¼/L W¼/W¾ LCh/L LCh/LPl LPl/WPl*	*P L W½ W¼ W¾ LCh WPl P/L W¼/L W¼/W¾ L/T½ LCh/LPl*	*W½/L W¼/L W¼/W¾*	*LPl WPl W½/L W¼/L W¼/W¾ L/T½ LCh/LPl LPl/WPl*		
PEL	*P L W½ W¼ W¾ LCh LPl WPl W½/L L/T½ LCh/L LCh/LPl*	*P L W½ W¼ W¾ LCh LPl WPl W½/L W¾/L W¼/W¾ L/T½ LCh/L LCh/LPl*	*W¼ W¼/L W¼/W¾*	*L/T½ LCh/LPl LPl/WPl*	*W¼ LPl LPl/WPl*	*W½ W¼ P/L W¼/L L/T½ LCh/LPl*	*P L W½ W¾ LCh LPl P/L W½/L LPl/WPl*	
COCH	*P L W½ W¼ W¾ LCh LPl WPl P/L W½/L W¼/L W¾/L L/T½ LCh/L LCh/LPl LPl/WPl*	*P L W½ W¼ W¾ LCh LPl WPl P/L W½/L W¼/L W¾/L L/T½ LCh/L LCh/LPl*	*P L W½ W¼ W¾ LCh LPl WPl P/L W½/L W¾/L L/T½ LCh/LPl*	*P L W½ W¼ W¾ LCh LPl WPl P/L W½/L W¼/L W¾/L L/T½ LPl/WPl*	*P L W½ W¾ L/T½ LCh/L*	*P L W½ W¼ W¾ LCh LPl WPl L/T½ LCh/L LCh/LPl*	*P L W½ W¼ W¾ LCh WPl W½/L W¼/L W¾/L W¼/W¾ L/T½ LCh/L*	*P L W½ W¼ W¾ LCh LPl WPl P/L W½/L W¼/L W¾/L LPl/WPl*
