# Supplementary material for: Taxonomic Significance of Seed Morphology in Veronica L. (Plantaginaceae) Species from Central Europe
Source: Plants (Basel). 2021 Dec 28;11(1):88. doi: 10.3390/plants11010088 (PMC8747532; doi:10.3390/plants11010088)
Supplement: Supplementary file 1 [file plants-11-00088-s001.zip › Table S2.pdf]

Table S2. The number of characters differing pairs of twenty-nine species of genus *Veronica* L. of nine subgenera; the result of the post-hoc Dunn's test. Subgenera abbreviations as in Table 3.

**PSEUD**	1	*V. longifolia*																												
	2	*V. spicata*	0																											
**BEC**	3	*V. anagalis-aq.*	0	0																										
	4	*V. beccabunga*	0	1	0																									
	5	*V. catenata*	0	0	0	0																								
	6	*V. peregrina*	0	0	0	1	0																							
	7	*V. serpyllifolia*	0	0	0	1	0	0																						
**VER**	8	*V. aphylla*	2	4	5	3	0	3	1																					
	9	*V. montana*	8	10	9	9	7	11	8	3																				
	10	*V. officinalis*	1	4	5	3	0	5	1	0	6																			
	11	*V. scutellata*	5	8	7	6	4	7	5	0	0	0																		
	12	*V. urticifolia*	1	1	5	1	0	1	0	0	4	0	0																	
**CHAM**	13	*V. arvensis*	4	3	5	5	2	3	2	4	9	5	8	4																
	14	*V. chamaedrys*	7	9	9	8	5	8	6	1	4	3	3	1	2															
	15	*V. dillenii*	8	9	11	10	0	9	3	0	5	1	1	0	1	0														
	16	*V. verna*	2	1	4	1	0	1	1	0	5	2	5	0	0	1	0													
**PEN**	17	*V. austriaca*	11	11	11	9	9	12	10	0	0	3	3	0	10	0	0	5												
	18	*V. teucrium*	0	2	6	4	0	1	0	0	1	0	0	0	0	0	0	0	0											
**STEN**	19	*V. fruticans*	7	9	8	7	4	10	7	0	2	0	0	0	10	3	2	6	2	0										
**POC**	20	*V. agrestis*	12	11	14	10	9	14	10	5	1	12	6	6	11	5	8	11	1	1	7									
	21	*V. filiformis*	1	1	6	3	0	2	0	0	4	1	2	1	0	0	0	1	0	0	2	2								
	22	*V. opaca*	13	10	13	10	7	13	8	2	0	7	4	1	7	0	4	8	3	0	5	0	0							
	23	*V. persica*	11	10	14	13	7	11	9	6	3	11	6	6	9	4	6	7	3	0	9	3	0	0						
	24	*V. polita*	8	9	12	11	4	10	4	2	6	4	5	1	4	2	2	3	5	0	6	6	1	2	7					
**PEL**	25	*V. praecox*	4	2	6	4	1	3	0	0	6	1	5	1	4	2	2	3	3	0	6	8	0	6	10	2				
	26	*V. triphyllos*	5	7	7	7	3	6	4	0	3	1	0	0	3	2	1	1	1	0	0	4	1	1	5	0	0			
**COCH**	27	*V. hederifolia*	15	14	15	12	9	16	14	9	0	11	7	8	13	7	10	12	1	4	10	3	6	5	7	9	10	6		
	28	*V. sublobata*	15	14	13	11	8	15	13	9	0	10	10	9	15	9	11	13	2	4	9	2	6	8	10	9	11	4	0	
	29	*V. triloba*	10	10	10	9	8	11	10	2	0	5	0	3	8	2	3	4	1	0	0	0	1	1	0	3	4	1	0	0
			1	*2*	*3*	*4*	*5*	*6*	*7*	*8*	*9*	*10*	*11*	*12*	*13*	*14*	*15*	*16*	*17*	*18*	*19*	*20*	*21*	*22*	*23*	*24*	*25*	*26*	*27*	*28*
			**PSEUD**			**BEC**				**VER**					**CHAM**			**PEN**		**STEN**	**POC**					**PEL**		**COCH**	
