# Supplementary material for: Taxonomic Significance of Seed Morphology in Veronica L. (Plantaginaceae) Species from Central Europe
Source: Plants (Basel). 2021 Dec 28;11(1):88. doi: 10.3390/plants11010088 (PMC8747532; doi:10.3390/plants11010088)
Supplement: Supplementary file 1 [file plants-11-00088-s001.zip › Table S3.pdf]

Table S3. Comparisons of seed size of *Veronica* species analysed in the present study and by Kulpa [29], Martinez-Ortega & Rico [30], and Hassan & Khalik [35]; bold – greatest values for the given species; italics – smallest values for the given species.

		**present study**							**Kulpa [26]**			**Hassan & Khalik [33]**				**Martinez-Ortega & Rico [27]**					
		L			W			T	L	W	T	L		W		L			W		
		M	Min	Max	M	Min	Max	M	M	M	M	Min	Max	Min	Max	M	Min	Max	M	Min	Max
**COCH**	*V. hederifolia*	2.16	1.10	2.60	1.82	1.28	2.33	1.27	**2.61**	**2.26**	**1.50**	*1.70*	*2.00*	2.00	2.30	-	-	-	-	-	-
	V. triloba	2.40	2.16	2.60	2.14	1.98	2.33	1.30	-	-	-	-	-	-	-	-	-	-	-	-	-
	V. sublobata	2.38	1.67	2.83	2.07	1.45	2.64	1.48	-	-	-	-	-	-	-	-	-	-	-	-	-
**CHAM**	*V. chamaedrys*	*1.27*	*0.81*	*1.55*	1.00	0.64	1.25	0.34	1.39	1.11	0.31	*1.20*	*1.50*	0.80	1.00	1.37	1.05	1.71	0.95	0.69	1.31
	V. arvensis	1.03	0.63	1.32	0.71	0.51	0.91	0.27	1.00	0.69	0.26	-	-	-	-	-	-	-	-	-	-
	V. verna	1.06	0.77	1.33	0.76	0.57	0.91	0.25	1.05	0.77	0.21	**1.00**	**1.50**	0.70	0.90	-	-	-	-	-	-
	V. dilleni	1.21	0.65	1.58	0.94	0.43	1.27	0.29	1.25	0.99	0.24	-	-	-	-	-	-	-	-	-	-
**PEN**	*V. austriaca*	1.47	0.88	2.00	1.22	0.74	1.55	0.41	**1.60**	**1.36**	0.35	1.40	1.80	1.20	1.60	*0.73*	*0.55*	*0.91*	*0.41*	*0.40*	*0.44*
	V. teucrium	*1.28*	*1.07*	*1.47*	*0.94*	*0.73*	*1.10*	0.35	1.48	1.36	0.30	-	-	-	-	**1.75**	**1.48**	**2.07**	1.23	1.11	1.48
**STEN**	*V. fruticans*	1.32	0.99	1.58	1.09	0.82	1.26	0.30	1.26	1.04	0.23	1.20	1.50	1.00	1.20	*1.19*	*0.90*	*1.34*	*0.91*	*0.69*	*1.09*
**VER**	*V. urticifolia*	1.13	0.95	1.35	0.87	0.67	1.00	0.21	1.17	0.93	0.19	*0.70*	*1.00*	0.70	0.90	1.06	0.90	1.24	0.87	0.58	1.05
	V. aphylla	1.21	0.98	1.40	0.94	0.71	1.15	**0.27**	1.14	0.96	0.19	1.20	1.50	1.00	1.20	1.25	1.00	1.64	1.00	0.80	1.17
	V. officinalis	1.10	0.90	1.33	0.88	0.71	1.04	0.27	1.01	0.78	0.24	0.80	1.20	0.70	0.90	**1.19**	**0.80**	**1.64**	0.92	0.33	1.21
	V. montana	2.04	1.30	2.45	**1.62**	1.12	2.00	0.36	2.00	1.23	0.29	-	-	-	-	1.95	1.53	2.47	1.66	1.13	2.07
	V. scutellata	1.40	0.83	1.63	1.11	0.90	1.40	0.14	1.35	1.10	0.20	-	-	-	-	**1.57**	**1.24**	**1.92**	1.18	0.60	1.49
**PEL**	*V. praecox*	1.10	0.80	1.40	0.84	0.56	1.10	0.60	1.08	0.79	0.51	1.00	1.30	0.50	0.90	-	-	-	-	-	-
	V. triphyllos	1.31	0.84	1.88	1.02	0.69	1.57	0.62	**1.58**	**1.21**	0.63	-	-	-	-	-	-	-	-	-	-
**POC**	*V. agrestis*	1.75	1.19	2.10	1.29	0.83	1.61	0.86	1.60	1.28	0.78	1.40	1.90	1.00	1.50	-	-	-	-	-	-
	V. polita	1.28	0.86	1.71	0.90	0.58	1.20	0.66	1.23	0.96	0.60	0.90	1.60	0.80	1.30	-	-	-	-	-	-
	V. opaca	1.50	0.88	2.22	1.10	0.47	1.60	0.68	**1.64**	**1.27**	0.69	-	-	-	-	-	-	-	-	-	-
	V. persica	1.64	1.29	2.09	1.11	0.80	1.53	0.71	1.68	1.21	0.74	**1.40**	**2.30**	0.85	1.60	-	-	-	-	-	-
	V. filiformis	1.25	1.09	1.45	0.90	0.72	1.00	0.51	**1.41**	**1.08**	0.56	-	-	-	-	-	-	-	-	-	-
**BEC**	*V. serpyllifolia*	0.82	0.65	0.97	0.56	0.44	0.67	0.20	0.85	0.60	0.21	*0.50*	*0.57*	0.50	0.60	**0.96**	**0.78**	**1.13**	**0.67**	**0.53**	**0.98**
	V. beccabunga	0.59	0.43	0.74	0.47	0.32	0.57	0.23	0.52	0.39	0.24	*0.50*	*0.55*	*0.30*	*0.40*	-	-	-	-	-	-
	V. anagallis-aquatica	0.51	0.33	0.72	0.36	0.25	0.54	0.22	0.52	0.40	0.23	**0.50**	**0.80**	0.40	0.55	-	-	-	-	-	-
	V. catenata	0.62	0.50	0.71	**0.47**	**0.35**	**0.56**	**0.27**	0.54	0.38	0.20	0.60	0.65	0.30	0.35	-	-	-	-	-	-
	V. peregrina	0.74	0.51	1.01	0.45	0.31	0.58	0.24	0.75	0.44	0.15	**1.00**	**1.50**	0.50	0.55	-	-	-	-	-	-
**PSEUD**	*V. longifolia*	0.73	0.52	0.88	0.53	0.41	0.64	0.22	**0.92**	**0.67**	0.23	-	-	-	-	-	-	-	-	-	-

V. spicata	0,63	0,47	0,96	0,42	0,31	0,56	0,20	**0,88**	**0,60**	**0,29**	0,80	1,10	0,50	0,65	-	-	-	-	-	-
