# Supplementary material for: Taxonomic Significance of Seed Morphology in Veronica L. (Plantaginaceae) Species from Central Europe
Source: Plants (Basel). 2021 Dec 28;11(1):88. doi: 10.3390/plants11010088 (PMC8747532; doi:10.3390/plants11010088)
Supplement: Supplementary file 1 [file plants-11-00088-s001.zip › Table S4.pdf]

Table S4. The list of herbarium specimens of twenty-nine *Veronica* L. species used in the study.

No.	Subgenus	Species	Herbarium	Sheet no.	Date	Collector	Locality
1	*Pocilla*	*V. persica*	BYDG	1179.F.	12.05.1991	K. Lach	Ślesin, Nakło district, meadow
3	*Pocilla*	*V. persica*	BYDG	1179.D	01 07 1987	E. Krasicka-Korczyńska	Starogard Gdański, clay field
9	*Pocilla*	*V. polita*	BYDG	1180. A	17.07.1993	M. Korczyński	Bydgoszcz, Zofin, field with rye
10	*Pocilla*	*V. polita*	BYDG	1180. D	15.08.1987	E. Krasicka-Korczyńska	Rudy, Bydgoszcz, field
11	*Pocilla*	*V. agrestis*	BYDG	1181	13.05.1985	E. Krasicka-Korczyńska	Rudy, Bydgoszcz, field
15	*Pocilla*	*V. opaca*	BYDG	1182	15.08.1991	E. Krasicka-Korczyńska	Bydgoszcz – Łęgnowo, field of cabbage
21	*Pocilla*	*V. persica*	TRN	s.n.	08.1892	Scholz	Kwidzyń
22	*Pocilla*	*V. persica*	TRN	s.n.	18.07.1884	H. Klingraft	Chmielno, Kartuzy
23	*Pocilla*	*V. persica*	TRN	s.n.	17.07.1884	H. Klingraft	Gleszembowo, Kartuzy
24	*Pocilla*	*V. persica*	TRN	s.n.	8.08.1885	Schwetz	Lnianno
25	*Pocilla*	*V. persica*	TRN	s.n.	10.1876	C. J. Klingraft	Polaszki
26	*Pocilla*	*V. persica*	TRN	s.n.	5.08.1883	Peil	Grudziądz
27	*Pocilla*	*V. persica*	TRN	s.n.	01 07 1904	Lange, H. Preuβ	Luboń
28	*Pocilla*	*V. agrestis*	TRN	s.n.	1.07.1886	A. Freirhel	Polaszki
29	*Pocilla*	*V. agrestis*	TRN	s.n.	29.08.1883	Kalmuss	Elbląk
30	*Pocilla*	*V. agrestis*	TRN	s.n.	07.1880	C. Lűtzow	Kartuzy
31	*Pocilla*	*V. agrestis*	TRN	s.n.	4.11.1893	Scholz	Toruń
32	*Pocilla*	*V. agrestis*	TRN	s.n.	09.1896	C. Lűtzow	Gdańsk, Oliwa
33	*Pocilla*	*V. polita*	TRN	s.n.	25.04.1826	Klinsmann	Gdańsk
35	*Pocilla*	*V. polita*	TRN	s.n.	27.04.1887	Scholz	Kwidzyn
36	*Pocilla*	*V. polita*	TRN	s.n.	1854	Krause	Wałcz

37	*Pocilla*	*V. polita*	TRN	s.n.	01 06 1906	Scholz	Rozpędziny near Kwidzyn
38	*Pocilla*	*V. opaca*	TRN	s.n.	07.1895	Scholz	Kwidzyn
39	*Pocilla*	*V. opaca*	TRN	s.n.	-	C. J. Klingraft	Polaszki near Kwidzyn
41	*Pocilla*	*V. opaca*	TRN	s.n.	09.1855	C. J. Klingraft	Stuhm, Polaszki
42	*Pocilla*	*V. opaca*	TRN	s.n.	1892	C. Lűtzow	Lębork
44	*Pocilla*	*V. opaca*	TRN	s.n.	23.08.1975	L. Rutkowski	Kwidzyn, loamy-sandy terrain along the road
47	*Cochlidiosperma*	*V. hederifolia*	TRN	s.n.	1981.05.20	D. Osińska	Otłoczyn, near Toruń, Salici-Populetum on Tążyna
48	*Cochlidiosperma*	*V. hederifolia*	TRN	s.n.	1964.05.14	M. Ceynowa-Giełdon, W. Gugnacka	Brześć Kujawski, Włocławek district
49	*Pseudolysimachium*	*V. longifolia*	TRN	s.n.	29.07.1970	J. Kotowicz, E. Kozioł	Śląsk Dolny, Ścinawa, Wołów district, the bank of the Odra River - on the edge of damp willow thickets
50	*Pseudolysimachium*	*V. spicata*	TRN	s.n.	25.07.1966	Rejewski	Słup-Młyn, gravel pit
52	*Chamaedrys*	*V. chamaedrys*	TRN	s.n.	10.07.1992	M. Bartkowiak	Mogilno, pine forest
53	*Chamaedrys*	*V. chamaedrys*	TRN	s.n.	17.07.1978	H. Dembińska	Słupsk district, Niedźwiady, near Lake Babinek
54	*Veronica*	*V. officinalis*	TRN	s.n.	15.06.1972	Zielski	Rosochy, Zbiczno, near Brodnica, oak and pine forest
55	*Veronica*	*V. officinalis*	TRN	s.n.	07.07.1964	Rejewski	Chełmno, oak and pine forest near Czernlewo
56	*Veronica*	*V. officinalis*	TRN	s.n.	27.06.1969	Trzebiatowski	Glinowo
57	*Veronica*	*V. montana*	TRN	s.n.	17.06.1966	Rejewski	Grudziądz, Mędrzycki Forest, gully on the slope of Łasinka, Tilio cordatae-Carpinetum betuli
58	*Veronica*	*V. montana*	TRN	s.n.	23.07.1975	E. Filipowska, F. Ludera	Bielsko-Biała, Uroczysko Cisowa in Monte Łazek
62	*Veronica*	*V. scutellata*	TRN	s.n.	1970.06	W. Gugnacka	Bukowa Primeval Forest, mid-forest swamp
63	*Veronica*	*V. scutellata*	TRN	s.n.	25.07.1966	Rejewski	Chojnice district, lake approx. 1 km from Swornigacie
64	*Beccabunga*	*V. beccabunga*	TRN	s.n.	08.07.1965	Rejewski	Chojnice district, Kiedrowickie Lake, near Lipnica
66	*Beccabunga*	*V. anagalis-aquatica*	TRN	s.n.	30.07.1958	Rejewski	near Łazin, Grudziądz distric, meadows and thickets
67	*Beccabunga*	*V. anagalis-aquatica*	TRN	s.n.	10.07.1979	B. Borka ? (zdj 626)	Toruń, Rubinkowo, Vistula Valley, shore of reservoir no.1

68	*Pocilla*	*V. arvensis*	TRN	s.n.	1970.06.18	Zielski	Wrocki, Golub-Dobrzyń district
69	*Pocilla*	*V. arvensis*	TRN	s.n.	1962.06.14	M. Rejewski	Szubin district, field near Lubostroń
72	*Chamaedrys*	*V. dillenii*	TRN	s.n.	1978.05.25	M. Ceynowa-Giełdon, W. Gugnacka	Toruń, Bielawy, at the edge of dry pine forest by Szosa Lubicka (road to Lubicz)
73	*Chamaedrys*	*V. dillenii*	TRN	s.n.	1969.06.06	Zielski	Cieszyny, Golub-Dobrzyń district
74	*Pellidosperma*	*V. triphyllos*	TRN	s.n.	1959.04.18	M. Rejewski	Suchatówki, Inowrocław district, on the lake
75	*Pellidosperma*	*V. triphyllos*	TRN	s.n.	1965.05.26	M. Rejewski	Toruń, around the airport
76	*Pellidosperma*	*V. praecox*	TRN	s.n.	1965.06.22	M. Ceynowa	Ryczkowo, Toruń district
77	*Stenocarpon*	*V. fruticans*	TRN	s.n.	12.06.1983	W. Gugnacka	Obłazowa (670 meters above sea level), Podhale, Bialka-Breakthrough
78	*Chamaedrys*	*V. verna*	KRA	358032	2002.06.15	M. Nobis	Mostki near Suchedniów, NE of Kielce, sandy shore of a lowered reservoir
79	*Chamaedrys*	*V. verna*	KRA	378672	2004.08.03	M. Nobis	Zalesie, near Bujak (approx. 15 km S of Radom), sandy edge of a farmland
80	*Chamaedrys*	*V. verna*	KRA	366111	2002.05.05	M. Nobis	Wąchoch, slope
81	*Chamaedrys*	*V. verna*	KRA	366129	2002.06.25	M. Nobis	Tychów Nowy, turf on sands
82	*Chamaedrys*	*V. verna*	KRA	396034	2005.06.02	M. Nobis	Krępianka river valley, around Solec nad Wisłą, grassland on calcareous sands
83	*Veronica*	*V. urticifolia*	KRA	248300	26.08.1967	S. Pelc	Pieniny, Ligarki, on the rocks with *Neckera crispa*, in the sycamore forest, exp. S-E
84	*Veronica*	*V. urticifolia*	KRA	248302	17.08.1968	S. Pelc	Dolina Dunajca, section Łąkcica-Stary Sącz, Biedroniówka, sand rocks on the Dunajec River, above the bridge in Jaworsko, right bank
85	*Veronica*	*V. urticifolia*	KRA	382022	26.05.2005	K. Stawowczyk	Beskid Sądecki - Pasmo Radziejowej - tatry Brzyńskie, approx. 400 m above sea level, in a deciduous forest
88	*Pellidosperma*	*V. triphyllos*	KRA	404740	2009.05.04	M. Wagda	Poj. Myśliborskie, Gryżyno, the shore of an old gravel pit
89	*Pellidosperma*	*V. triphyllos*	KRA	409904	2008.04.23	A. Trojecka-Brzezińska	Wzgórze Opoczyńskie (part E), Bedlenko, farmland
90	*Pellidosperma*	*V. triphyllos*	KRA	384560	2008.05.10	K. Stawowczyk	Beskid Sądecki – Pasmo Radziejowej - Barcice Dominików, approx. 240 m above sea level, on a fallow land with advanced succession
91	*Cochlidiosperma*	*V. triloba*	KRA	393736	2009.05.06	M. Bielecki	Włoszczowska Niecka Mesoregion, Czarna - Zastaw, edge of an arable field

92	*Pentasepalae*	*V. teucrium*	KRA	358440	24.06.2006	M. Nobis	Piekary near Krakow, xerothermic thickets at the limestone rock by the Vistula riverbed
93	*Pentasepalae*	*V. teucrium*	KRA	357992	28.07.2006	A. Nobis, M. Nobis	on the SE from the village of Bębło near Jerzmanowice, on the left side of the Krakow-Olkusz road, xerothermic grassland on a limestone outlier
96	*Cochlidiosperma*	*V. sublobata*	KRA	305663	1998.05.05	K. Towpasz, H. Trzcińska-Tacik	Ostrów, broadleaved forest
98	*Cochlidiosperma*	*V. sublobata*	KRA	368267	1998.05.01	H. Trzcińska-Tacik	Płaskowyż Proszowicki, Góry Sieradzkie near Ostrów, forest in the western part near the road to Wierzchowice
99	*Cochlidiosperma*	*V. sublobata*	KRA	378853	1983.04.30	H. Trzcińska-Tacik	Puszcza Niepołomicka, Chobot, broadleaved forest
100	*Cochlidiosperma*	*V. sublobata*	KRA	378854	1983.04.30	H. Trzcińska-Tacik	Puszcza Niepołomicka
101	*Cochlidiosperma*	*V. sublobata*	KRA	376811	1997.06.05	H. Trzcińska-Tacik	Brzezowo - forest on the slope of Zuchowa Góra by the Zalew Dobrzycki, from the top, from the side of usable meadows, W side, forest on the slope
102	*Pseudolysimachium*	*V. spicata*	KRA	352043	03.09.2005	M. Podgórska	Garb Gielniowski, Kurzacze near Rzepycha, dry turf
105	*Pseudolysimachium*	*V. spicata*	KRA	356136	07_2000	R. Piwowarczyk	Wólka Bałtowska - a village 2 km N from Bałtów, xerothermic grassland
106	*Pseudolysimachium*	*V. spicata*	KRA	338593	30.07.1999	P. Bochenek, M. Zarzyka-Rynka	Pieniny, Wąwóz Leśnickiego Potoku, rocky grasslands
109	*Beccabunga*	*V. serpyllifolia*	KRA	323047	11.08.2002	M. Nobis	on the slope of Dolina Kamienna - Kol. Podlesie near Brody Iłżeckie, turf on the slope
110	*Beccabunga*	*V. serpyllifolia*	KRA	391115	28.05.2007	B. Gutkowska	Konieczkowa, forest
111	*Beccabunga*	*V. serpyllifolia*	KRA	395116	12.06.2008	M. Bielecki	Włoszczowska Niecka Mesoregion, Michałów, meadow (close to the tracks)
113	*Veronica*	*V. scutellata*	KRA	259307	16_08.1994	W. Bartoszek	Western Carpathians - Beskid Makowski (Średni) - E part of Pcim (Myślenice district), a swampy meadow by a gravel road at the foot of the escarpment (slopes of the Raba valley), approx. 335 m above sea level
114	*Veronica*	*V. scutellata*	KRA	268843	03.09.1965	D. Fijałkowski	Żabce near Międzyrzecze, Lublin region, meadow
115	*Veronica*	*V. scutellata*	KRA	310680	08.07.1999	M. Zarzyka	Beskid Niski, Wisłoka valley, Radocyna, mlak
116	*Veronica*	*V. scutellata*	KRA	324605	26.07.2007	M. Kozak	Piaski (W from Działoszyn), margin of wet meadow
117	*Veronica*	*V. scutellata*	KRA	316697	12.08.1980	K. Towpasz	Pogórze Strzyżowskie, Dobrzechów, shore of the fish pond
118	*Pellidosperma*	*V. praecox*	KRA	207833	2001.06.07	H. Trzcińska-Tacik	Płaskowyż Proszowicki, around Proszowice-Gniazdowice, in the

							gaps of grassland with Stipa capillata, to the E from the quarry
119	*Pellidosperma*	*V. praecox*	KRA	347988	2008.05.25	A. i M. Nobis	Smogonów, near Busko-Zdrój, field roadside
120	*Pellidosperma*	*V. praecox*	KRA	347969	2008.05.25	A. i M. Nobis	Żerniki Górne, near Busko-Zdrój, farmland
121	*Pellidosperma*	*V. praecox*	KRA	361667	1997.05.23	H. Trzcińska-Tacik	Stawiany near Pińczów, the field between the gypsum hills
122	*Pellidosperma*	*V. praecox*	KRA	365375	1995.05.02	H. Trzcińska-Tacik	Gniazdowice near Proszowice, steep slope on gypsum rock
123	*Pocilla*	*V. polita*	KRA	317039	18.09.1979	K. Towpasz	Przeczyca, Pogórze Strzyżowskie, on the field in potatoes
124	*Pocilla*	*V. polita*	KRA	324657	07.07.2001	M. Nobis	Przegórze Iłżeckie, Mądralów near Wierzbica, calcareous farmland
126	*Pocilla*	*V. polita*	KRA	259719	28.05.2004	Z. Głowacki	Tokarnia, Chęciny district, fallow
127	*Pocilla*	*V. polita*	KRA	242640	02.09.1998	K. Wilk	Tuczempy, the edge of the riparian forest in the San valley, 180 m above sea level
128	*Beccabunga*	*V. peregrina*	KRA	293940	1987.08.29	M. Zając	Kotlina Oświęcimska: Bronów, the bottom of a dry pond
129	*Beccabunga*	*V. peregrina*	KRA	301011	2006.10.04	W. Bartoszek	Western Carpathians – Kotlina Żywiecka, Żywiec, downstream of Moszczanica (Żywiec district), muddy shores of the Żywieckie Lake
130	*Beccabunga*	*V. peregrina*	KRA	357776	2009.09.25	A. i M. Nobis	Osiek / Łęki, approx. 200 m E from the main road Oświęcim - Kęty
131	*Beccabunga*	*V. peregrina*	KRA	349979	2007.06.08	M. Podgórska	Korytków, between Końskie and Gielniów, bottom of the breeding pond
134	*Pocilla*	*V. opaca*	KRA	305662	21.06.1996	K. Towpasz, H. Trzcińska-Tacik	Posądza near Proszowice, farmland
135	*Pocilla*	*V. opaca*	KRA	367540	30.08.2000	H. Trzcińska-Tacik	Tempaczów-Rędziny, field of root crops
136	*Pocilla*	*V. opaca*	KRA	375723	06.09.1997	H. Trzcińska-Tacik	Jakuszowice near Kazimierz Wlk., field in potatoes
137	*Pocilla*	*V. opaca*	KRA	376072	04.07.2004	M. Nobis	Pętkowice near Bałtów, calcareous farmland
138	*Pocilla*	*V. opaca*	KRA	402716	08.09.1998	H. Trzcińska-Tacik	Cieszkowy, Płaskowyż Proszowicki, in the potato field in the black soil
139	*Veronica*	*V. officinalis*	KRA	383679	16.07.2006	K. Kozłowska	Kolonia Chełmiec, Pogórze Złotoryjskie (Western Sudetes), forest in the top part of Zamokowa
140	*Veronica*	*V. officinalis*	KRA	366887	14.08.2000	M. Nobis	Pakosław near Iłża, forest edge
141	*Pocilla*	*V. persica*	KRA	322511	20.04.2002	M. Nobis	Iłża, roadside

142	*Pocilla*	*V. persica*	KRA	353745	13.07.2004	R. Piwowarczyk	Wesołówka, farmland
143	*Pocilla*	*V. persica*	KRA	335016	10.08.2005	M. Hawro	Sarzyna, farmland
144	*Pocilla*	*V. persica*	KRA	333211	05.07.2005	A. Nobis	between Sieniawa and Ubieszyn, E part. Kotlina Sandomierska, farmland on the San river
145	*Pocilla*	*V. persica*	KRA	333071	20.08.2004	A. Michalewska	On the W of Kuryłówka village, the Lower San Valley, farmland on the San river
146	*Veronica*	*V. officinalis*	KRA	395089	20.09.2007	M. Bielecki	Włoszczowska Niecka Mesoregion, near Zaprzeczki, pine forest
147	*Veronica*	*V. officinalis*	KRA	403731	12.07.2005	D. Kempa, W. Bartoszek	Western Carpathians - Beskid Makowski (Sredni) - Middle part, Zachełmna (near Budzów, Suski district), meadow
148	*Veronica*	*V. montana*	KRA	82070	27.07.1967	K. Towpasz	Beskid Wyspowy - Jasień, slope N, 800 m above sea level, fir and beech forest
150	*Veronica*	*V. montana*	KRA	107110	05.07.1981	M. Matyjaszkiewicz, H. Trzcińska-Tacik, J.Radwański	Lipowiec near Chrzanów, around Kraków, a broadleaf forest
153	*Pseudolysimachium*	*V. longifolia*	KRA	394622	26.08.2008	M. Bielecki	Włoszczowska Niecka Mesoregion, Komparzów, wet meadow
154	*Pseudolysimachium*	*V. longifolia*	KRA	266766	15.07.2000	J. Mitka	Kraków Swoszowice, the area taken under the motorway, from Kąpielowa Street, wet meadow
155	*Pseudolysimachium*	*V. longifolia*	KRA	276407	02.08.2005	B.Binkiewicz, K. Błaszczykiewicz	Hajnówek, The Puszcza Romincka, shore of the ditch
156	*Pseudolysimachium*	*V. longifolia*	KRA	324556	23.07.2002	M. Nobis	Kamienna valley, next to the tracks – The Staw Kunowski, meadow
157	*Cochlidiosperma*	*V. hederifolia*	KRA	370850	1974.05.18	H. Trzcińska-Tacik	Kraków-Pychowice
158	*Cochlidiosperma*	*V. hederifolia*	KRA	378331	1973.05.05	H. Trzcińska-Tacik	Ujazd village, around Krakow, field
159	*Cochlidiosperma*	*V. hederifolia*	KRA	377534	05.05.1996	H. Trzcińska-Tacik	The Płaskowyż Proszowicki, around Jakuszowice
160	*Cochlidiosperma*	*V. hederifolia*	KRA	380634	2005.05.02	K. Kozłowska	Leszczyna, Złotoryja district, deciduous forest
161	*Cochlidiosperma*	*V. hederifolia*	KRA	380685	2005.04.30	K. Kozłowska	Jerzmanice-Zdrój, Złotoryja district, roadside, forest
162	*Stenocarpon*	*V. fruticans*	KRA	171306	11.09.1956	E. Pauer	Gubałowskie hill, Roztoki, 865 m above sea level, gravel pit
163	*Stenocarpon*	*V. fruticans*	KRA	171304	25.07.1961	R. Rejchel	Western Tatras, around Spadorz, 1160 m above sea level, moist gravel
164	*Stenocarpon*	*V. fruticans*	KRA	138500	20.08.1986	A. Sendek	Tatrzański National Park, Przełęcz Iwaniówka - approach
165	*Stenocarpon*	*V. fruticans*	KRA	112811	19.06.1961	R. Rejchel	Western Tatras, Dolina Ku Dziurze, 975 m above sea level, rocks

							by the stream
166	*Stenocarpon*	*V. fruticans*	KRA	126356	02.09.1933	J Naus (???) fot 8084	Western Tatras, Dolina Za Bramką, on the river
168	*Pocilla*	*V. filiformis*	KRA	248316	15.06.1965	B. & L. Stuchlikowie	Pogórze Przemyskie
169	*Pocilla*	*V. filiformis*	KRA	332592	24.06.2004	A. Michalewska	Rędziny Sibigi near Kamionka Dln., meadow on the San
170	*Chamaedrys*	*V. dillenii*	KRA	366577	1995.07.08	H. Trzcińska-Tacik	around Krakow, Przeginia Narodowa, sandy
171	*Chamaedrys*	*V. dillenii*	KRA	370966	2004.06.26	H. Trzcińska-Tacik	Płaskowyż Proszowicki, Zagórzyce, sandy fallow land near the forest
172	*Chamaedrys*	*V. dillenii*	KRA	372241	2004.07.10	H. Trzcińska-Tacik	Suwalski PK, Smolniki
173	*Chamaedrys*	*V. dillenii*	KRA	371393	1977.05.25	H. Trzcińska-Tacik	okol. Dębiny, Nagoszyn-Wola, edge of the forest and fields
174	*Chamaedrys*	*V. dillenii*	KRA	362619	2004.06.26	H. Trzcińska-Tacik	Płaskowyż Proszowicki, Zagórzyce, sandy fallow land near the forest
175	*Chamaedrys*	*V. dillenii*	KRA	362623	2004.06.26	H. Trzcińska-Tacik	Wyżyna Częstochowska, Chełm, near Wolbrom, a grain field
176	*Chamaedrys*	*V. chamaedrys*	KRA	393138	15.06.2008	M. Bielecki	Włoszczowska Niecka Mesoregion, Włoszczowce - Podzamcze, meadow
177	*Chamaedrys*	*V. chamaedrys*	KRA	232506	07_1976	M. Łachańska	Mniszek, meadow
178	*Chamaedrys*	*V. chamaedrys*	KRA	279242	05.07.2000	I. Skrzynecka	Niecka Nidziańsko-Polaniecka, Smordyna, roadside
179	*Chamaedrys*	*V. chamaedrys*	KRA	322045	14.08.2003	M. Nobis	Małyszyn, Starachowice district, broadleaved forest
180	*Chamaedrys*	*V. chamaedrys*	KRA	380681	10.07.2005	K. Kozłowska	Jerzmanice-Zdrój,Złotoryja district, Kaczaw valley, riverside meadows
181	*Beccabunga*	*V. catenata*	KRA	362158	12.07.1961	H. Trzcińska	Dolina Dolnej Nidy, Piastrzec near Solec-Zdrój
182	*Beccabunga*	*V. catenata*	KRA	336068	23.08.2003	M. Zarzyka-Rynka	Nizina Nadwiślańska, the left bank of the Vistula River, Przemyków, terrace, watercourse
183	*Beccabunga*	*V. beccabunga*	KRA	350381	05.08.2006	M. Podgórska	Ruszkowice near Rzepicha, riverbank
184	*Beccabunga*	*V. beccabunga*	KRA	339685	21.06.2001	A. Michalewska	Dolina Mnikowska, Wyżyna Karkowsko-Częstochowska, along the stream
185	*Beccabunga*	*V. beccabunga*	KRA	379285	04.06.2005	H. Trzcińska-Tacik	on the E from Przeginia Narodowa, a wet place, bushes
186	*Beccabunga*	*V. beccabunga*	KRA	374113	04.06.1967	H. Trzcińska-Tacik, T. Tacik	Dolina Szreniawy, on the S-E from Słomniki, a depression with water, under the left edge of the valley
187	*Beccabunga*	*V. beccabunga*	KRA	380672	16.07.2006	K. Kozłowska	Kolonia Chełmiec, Pogórze Złotoryjskie (Sudety Zachodnie), in the stream

189	*Pentasepalae*	*V. austriaca*	KRA	396620	02.07.2004	M. Nobis	Podgrodzie near Ćmielów, at the steep edge of the Kamienna valley slope, on a limestone rock
190	*Pentasepalae*	*V. austriaca*	KRA	306659	14.07.1997	K. Towpasz	Płaskowyż Proszowicki, Przemęczanki, xerothermic turf on a slope
191	*Pentasepalae*	*V. austriaca*	KRA	359037	14.07.2003	R. Piwowarczyk	NW from Grójec, a steep loess slope
192	*Chamaedrys*	*V. arvensis*	KRA	232431	1983.05.15	P. Badek	Rokitnica, old gravel pit
193	*Chamaedrys*	*V. arvensis*	KRA	238368	1991. 06. 16	E. Pogan	Gdańsk-Oliwa, at the wall of the parking
194	*Chamaedrys*	*V. arvensis*	KRA	263687	2002.06	K. Kulpiński	near Myszków, pond
195	*Chamaedrys*	*V. arvensis*	KRA	257500	1963.06.14	St. Telc	Pogórze Cieszyńskie, Ustroń, gravel pit on the Vistula River
196	*Chamaedrys*	*V. arvensis*	KRA	240209	1969.06.16	St. Telc	Dolina Dunajca, Sromowce Niżne, dry gravel shaft
197	*Veronica*	*V. aphylla*	KRA	112807	03.06.1961	R. Rejchel	Tatry Zachodnie, Dolina Za Bramką, 920 m above sea level, by a stream
198	*Veronica*	*V. aphylla*	KRA	126174	08.09.1928	J. Walas	Babia Góra
199	*Veronica*	*V. aphylla*	KRA	374436	08.09.1990	H. Trzcińska-Tacik	Tatra Mountains, the pass between Kopas, over Dolina Jaworzynki, grassy turf, rocky place
200	*Beccabunga*	*V. anagalis-aquatica*	KRA	338189	09.07.2007	J. Żelazny, K. Kozłowska	Chełm by the Raba River, riverbank
202	*Beccabunga*	*V. anagalis-aquatica*	KRA	350422	23.08.2005	M. Podgórska	Majdów, near Skarżysko-Kamienna, a drainage ditch
203	*Beccabunga*	*V. anagalis-aquatica*	KRA	394748	20.08.2009	M. Bielecki	Włoszczowska Niecka Mesoregion, Rząbiec
204	*Beccabunga*	*V. anagalis-aquatica*	KRA	394744	15.10.2008	M. Bielecki	Włoszczowska Niecka Mesoregion, Bałków, silt at the bottom of a drained pond
205	*Beccabunga*	*V. anagalis-aquatica*	KRA	398756	01.08.2009	M. Wajda, K. Szurdak	Włoszczowska Niecka Mesoregion, Wolin 53 st 50', 14 st 37'' (fot. 8149), drainage ditch
206	*Pocilla*	*V. agrestis*	KRA	410026	26.05.2009	A. Trojecka-Porzezińska	Wzgórze Opoczyńskie, Ogonowice, farmland
207	*Pocilla*	*V. agrestis*	KRA	362345	06.07.2009	A. Nobis	Kulno near Leżajsk, Dolina Dolnego San, S end of the village of Kulno, farmland, potato cultivation
208	*Pocilla*	*V. agrestis*	KRA	314062	13.07.2006	M. Kozak	Kraków-Kościelniki, edge of the farmland
209	*Pocilla*	*V. agrestis*	KRA	323266	20.07.2004	M. Nobis	Wysoka Stara near Szydłowiec, farmland near the church
210	*Pocilla*	*V. agrestis*	KRA	330419	30.07.2003	A. Michalewska	Biszcza, moist farmland (in tobacco)
