# Supplementary figures and images for: Taxonomic Significance of Seed Morphology in Veronica L. (Plantaginaceae) Species from Central Europe
Source: Plants (Basel). 2021 Dec 28;11(1):88. doi: 10.3390/plants11010088 (PMC8747532; doi:10.3390/plants11010088)

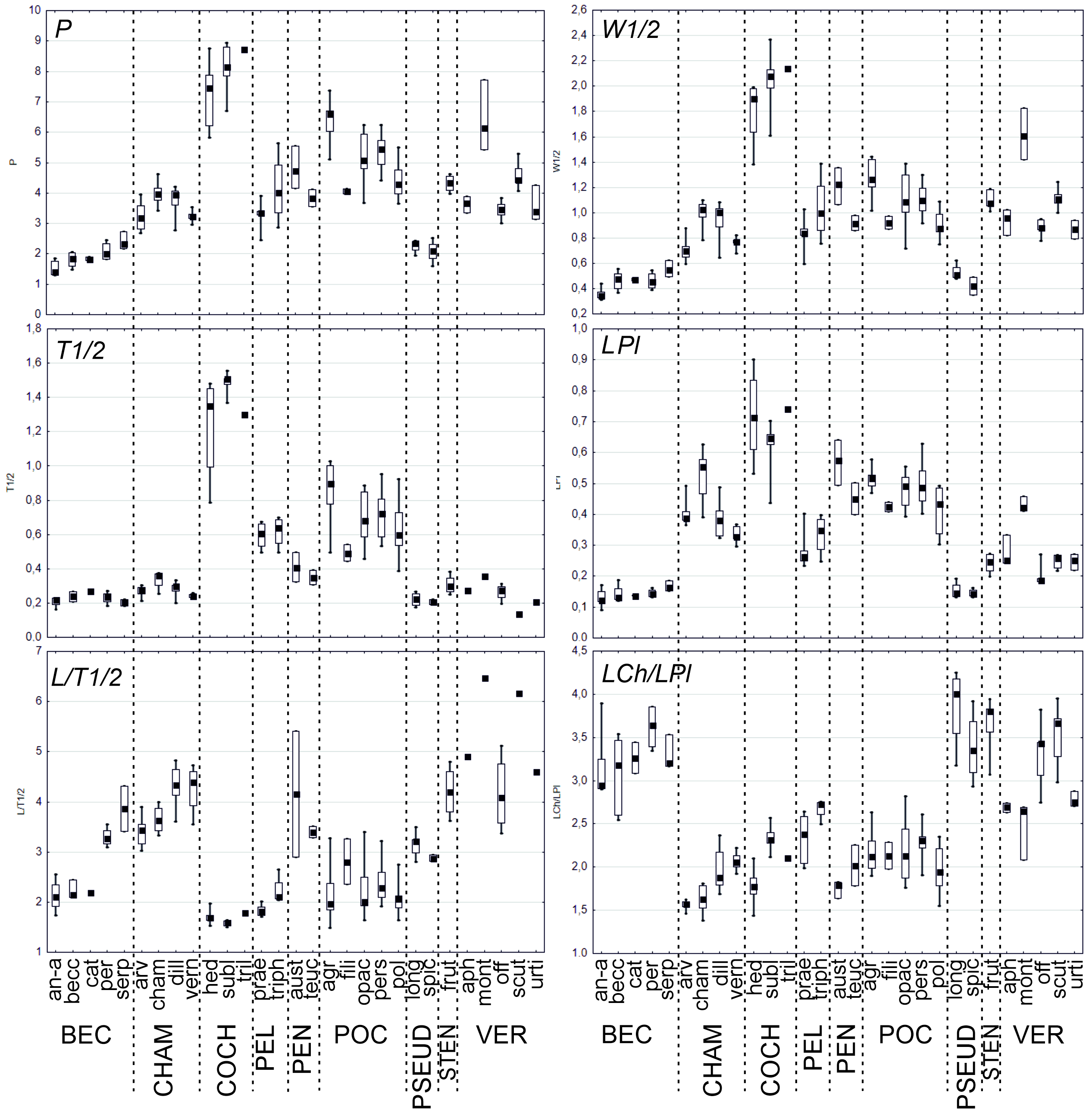

Supplement: Supplementary file 1 [file plants-11-00088-s001.zip › Fig. S1.tif]

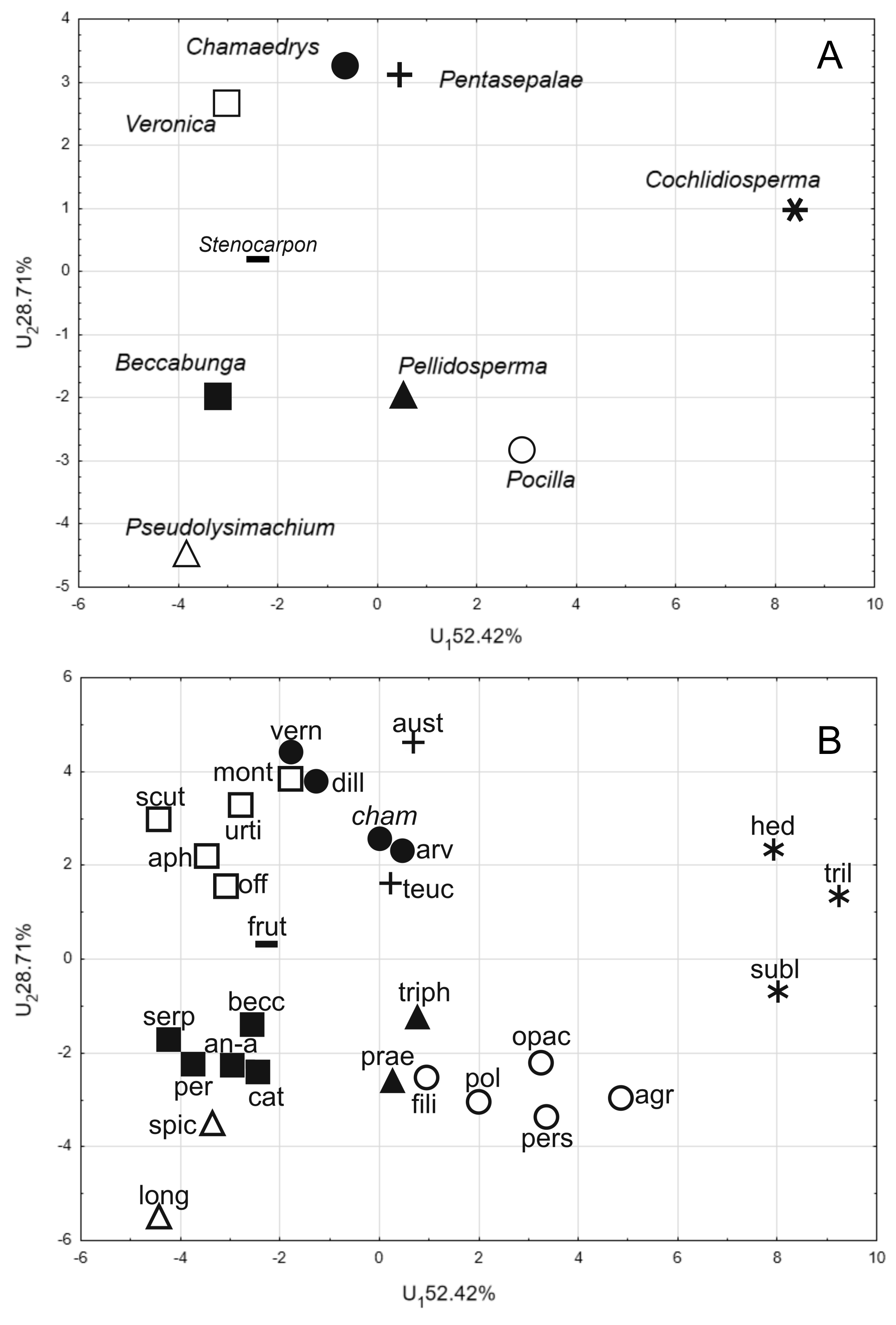

Supplement: Supplementary file 1 [file plants-11-00088-s001.zip › Fig. S2.tif]
